# Risk factor analysis of allergic rhinitis in 6–8 year-old children in Taipei

**DOI:** 10.1371/journal.pone.0249572

**Published:** 2021-04-02

**Authors:** Ciao-Lin Ho, Wei-Fong Wu

**Affiliations:** 1 Second Degree Bachelor of Science in Nursing, College of Medicine, National Taiwan University, Taipei, Taiwan, ROC; 2 Department of Pediatrics, Taipei City Hospital Ren-Ai Branch/Department of Allergy and Immunology, West Garden Hospital, Taipei, Taiwan, ROC; Norwegian Institute of Public Health, NORWAY

## Abstract

The incidence of allergic rhinitis (AR) has increased rapidly in Taiwan during the past 30 years; however, potential risk factors of AR have yet to be examined. The purpose of this study is to explore the prevalence, personal and environmental risk factors of rhinitis. A cross-sectional survey was conducted in 26418 first graders (6–8 years old) in Taipei with a response rate of 94.6% (24999/26418). Modified International Study of Asthma and Allergies in Childhood (ISAAC) questionnaires were completed by their parents or main caregivers. Logistic regression was used to examine possible personal and environmental (in early life and current) factors related to rhinitis. The prevalence of rhinitis in the past 12 months was 42.8% in 6–8 years old children. Multivariate logistic regression analysis for both males and females revealed that male gender, antibiotic use in first year of life, bronchiolitis before the age of two years, diagnosed asthma, and diagnosed eczema, having a cat the first year of life were associated with an increased risk of rhinitis. Having older siblings, on the other hand, may reduce the risk of rhinitis. Based on the present study, we may recommend less use of antibiotics the first year of life and not having a cat in the home in the child’s first year of life as preventive measures to reduce the risk of rhinitis. From the subgroup analysis, we can take preventive measures for the different risk factors of rhinitis and the severity of rhinitis in each subgroup.

## Introduction

Allergic rhinitis, the most common chronic pediatric disorder [1,2], is an inflammatory condition of the nasal mucosa in which the mucous membrane overreacts when exposed to allergens that are idle in normal subjects. This annoying problem is common in Taiwan with island climate. Non-allergic responses of nasal membrane is the main feature of AR, i.e., an increased response to normal stimulations that induce sneezing, nasal mucosa congestion (blocked nose), together with increased secretion (running nose). In severe condition, the patients may feel dizzy, headache, itchy at various parts (e.g., eyes, ears, face, throat, etc.) ear fullness, etc., which further cause other health problems and weaken one’s daily life performance (e.g., memory and normal thinking process). Common complications that come with AR include sinusitis, Eustachian tube dysfunction, olfactory dysfunction, sleep disorder, increase of headache frequency, and various problems caused by long term mouth breathing [3]. Not only the persisting or lifelong treatment of AR increases the psychological and economic burden of the patients, it also increases the overall medical cost of the nation [1,4].

The triggers of rhinitis are multifactorial, including personal and known environmental factors, which can lead to sensitization and later development of allergic diseases. Thus, examining the etiological factors of rhinitis is important for preventing and treating of this common disorder in children. Environmental factors inferred to have a stronger influence in the youngest age group and could help in determination of effective intervention measures [5]. Many studies had taken into consideration the field of personal and environmental factors of rhinitis. However, most studies in Taiwan only paid attention on the prevalence of rhinitis, and few large studies to explore the risk factors and the multiple environment interaction of rhinitis.

Therefore, this study aims to explore possible environmental and personal risk factors for rhinitis in 6–8 years old children in Taipei, Taiwan. The results of this study would aid in planning important policy-related prevention strategies for early intervention and treatment of rhinitis.

## Materials and methods

This study method was comprised of questionnaires includes brief description of symptoms and signs of rhinitis. The study was approved by the Institution Review Board of Taipei City Hospital (TCHIRB-961007-E) and provided written informed consent for the parents or guardians of the children. We approached 153 elementary schools in 12 administrative districts in Taipei for permission to conduct the questionnaire survey in first graders (aged 6- to 8-year-old) in 2007. The parents or guardians were not directly asked whether their children had rhinitis. Instead, they were first presented with a brief description of typical signs and symptoms of those allergic diseases and then filled out the questionnaires and the informed consent. To analyze these issues, we used questionnaires modified from the International Study of Asthma and Allergy in Childhood (ISAAC) questionnaire, to evaluate the prevalence and risk factors of childhood rhinitis in Taipei.

Questionnaires are to provide a framework for further etiological research into personal and environmental factors affecting rhinitis. Personal factors included gender, body mass index (BMI), type of delivery, antibiotic use in the first year of life, bronchiolitis before the age of two, birth weight, presence of older siblings, presence of younger siblings, breastfeeding, diagnosed asthma, and diagnosed eczema. Details of environmental factors include the following: cats in the first year of life, cats in the past 12 months, dogs in the first year of life, dogs in the past 12 months, as well as farm animals in the first year of life. The meaning of the variables is defined in Table 1.

**Table 1:** Definition of the variables for questionnaires.

Variable	The meaning
Gender	Male or female
BMI	Body mass index
Type of delivery	C/S: Caesarean section; NSD: Normal spontaneous delivery
Antibiotic use in the first year of life	In the first 12 months of life, did your child have any antibiotics?
Bronchiolitis before the age of two	Has your child bronchiolitis before the age of two?
Birth weight	What was the weight of your child when he/she was born?
Older siblings	How many older brothers and sisters does your child have?
Younger siblings	How many younger brothers and sisters does your child have?
Breast- feeding	Was your child breast- feeding <4 months or ≧4 months?
Diagnosed asthma	Has your child ever had asthma?
Diagnosed eczema	Has your child ever had eczema?
Cats in the first year of life	Did you have a cat in your home during the first year of your child’s life?
Cats in the past 12 months	In the past 12 months, have you had a cat in your home?
Dogs in the first year of life	Did you have a dog in your home during the first year of your child’s life?
Dogs in the past 12 months	In the past 12 months, have you had a dog in your home?
Farm animals in the first year of life	In your child’s first year of life did he / she have regular at least once a week contact with farm animals (e.g. cattle, pigs, goats, sheep or poultry)?

This study analyzed the risk factors for rhinitis ever and rhinitis the last 12 months. The subgroups included males and females, only males, only females, only rhinitis, rhinitis & asthma, rhinitis & eczema, mild rhinitis, moderate rhinitis, severe rhinitis, and rhinitis ever. The severity of rhinitis was divided into groups as mild rhinitis, moderate rhinitis, and severe rhinitis.

Sixteen independent variables in personal factors and environmental factors were included individually into the univariate Logistic regression analysis and were used to estimate odds ratios (OR) and 95% confidence intervals (CIs) for the association between personal factors, environmental factors and rhinitis symptoms in the past 12 months.

A p value < 0.05 was set to determine level of significance in the univariate Logistic regression analysis. Univariate influence was explored by comparing a logistic regression model that contains only one independent variable and one dependent variable. Next, the significant univariate relative to rhinitis were put into multivariate consideration. Multiple regression models were used to determine whether rhinitis was associated with the significant variables. Multiple regression models presented p<0.05 and the corrected value simultaneously. We corrected for multiple hypothesis testing by dividing the P-value of 0.05 by the effective number of test to obtain a new threshold.

All significant univariates of logistic regression (binary variables) were put into the linear regression model to obtain Tolerance or variance inflation factor (VIF) in collinearity analysis. If Tolerance < 0.1 or VIF > 10, it means that there is collinearity. The interaction analysis uses the general linear model to estimate the interaction of all significant univariates. The interaction effects are all at a significant level, indicating that there is an interaction.

The logistic regression was used to estimate link influence in a directed acyclic graph (DAG) for each potential causal effect in which the variable at the base of the arrow (‘cause’) was considered a covariate, and the variable at the head of the arrow (‘effect’) was considered the outcome or dependent variable. All statistical analyses were performed with IBM SPSS Statistics 22.

## Results

A total of 26,418 copies of questionnaire were distributed and 25,097 were returned (response rate = 94.6%) in Taiwan. A total of 24,999 completed questionnaires returned from 153 primary schools were transformed into digital format. The study subjects were 6- to 8-year old children, and the questionnaires with incomplete or bizarre data for analyzing were excluded from the sample to left 23,630. Table 2 demonstrates the number of participants with rhinitis/no rhinitis with regard to the personal and environmental variables for males and females, and Table 3 shows the results from the univariate and multivariate analyses (both personal and environmental factors combined) for males and females.

**Table 2:** The number of participants with rhinitis/no rhinitis with regard to the personal and environmental variables for males and females.

	No rhinitis (*n* = 12978)	rhinitis (n = 10652)
Variable	*n (%)*	*N*
Gender		
Male	6116(50.0)	6120 (50.0)
Female	6862 (60.2)	4532 (39.8)
BMI		
Overweight/ obesity	2950 (53.6)	2555 (46.4)
Underweight/ normal	7692 (53.7)	6632 (46.3)
Type of delivery		
C/S	4483 (54.2)	3787 (45.8
NSD	8241 (55.2)	6699 (44.8)
Antibiotic use in the first year of life		
Yes	1494 (41.9)	2072 (58.1)
No (including unknown)	11138 (57.2)	8322 (42.8)
Bronchiolitis before the age of two		
Yes	1801 (38.9)	2833 (61.1)
No (including unknown)	10844 (59.0)	7541 (41.0)
Birth weight		
Underweight (<2500 g)	806 (53.2)	708 (46.8)
Normal (≧2500 g)	10768 (54.0)	9166 (46.0)
Older siblings		
Yes	6878 (60.6)	4468(39.4)
No	5928 (49.3)	6093 (50.7)
Younger siblings		
Yes	4345 (51.6)	4075 (48.4)
No	8461 (56.6)	6486 (43.4)
Breast- feeding		
<4 months (including none)	10496 (55.0)	8598 (45.0)
≧4 months	2281 (54.5)	1903 (45.5)
Diagnosed asthma		
Yes	959 (31.2)	2117 (68.8)
No	11902 (58.6)	8412 (41.4)
Diagnosed eczema		
Yes	2970 (41.2)	4233 (58.8)
No	9582 (61.6)	5978 (38.4)
Cats in the first year of life		
Yes	257 (48.3)	275 (51.7)
No	12628 (55.1)	10303(44.9)
Cats in the past 12 months		
Yes	1318 (54.3)	1108 (45.7)
No	11555 (54.9)	9474 (45.1)
Dogs in the first year of life		
Yes	1457 (50.6)	1422 (49.4)
No	11413 (55.5)	9165 (44.5)
Dogs in the past 12 months		
Yes	1318 (54.3)	1108 (45.7)
No	11555 (54.9)	9474 (45.1)
Farm animals in the first year of life		
Yes	256 (47.1)	288 (52.9)
No	12614 (55.0)	10300 (45.0)

C/S: Caesarean section; NSD: Normal spontaneous delivery.

**Table 3:** The results from the univariate and multivariate analyses (both personal and environmental factors combined) for males and females.

	Univariate				Multivariate			
Variable	***P***	***OR***	***lower***	***upper***	***P***	***OR***	***lower***	***upper***
Gender								
Male	<0.001	1.52	1.44	1.60	<0.001	1.45	1.37	1.54
Female		1				1		
BMI								
Overweight/ obesity	.887	1.00	0.94	1.07				
Underweight/ normal		1						
Type of delivery								
C/S	.163	1.04	0.98	1.10				
NSD		1						
Antibiotic use in the first year of life								
Yes	<0.001	1.86	1.73	2.00	<0.001	1.34	1.23	1.45
No (including unknown)		1				1		
Bronchiolitis before the age of two								
Yes	<0.001	2.26	2.12	2.42	<0.001	1.68	1.55	1.81
No (including unknown)		1				1		
Birth weight								
Underweight (<2500 g)	.556	1.03	0.93	1.15				
Normal (≧2500 g)		1						
Older siblings								
Yes	<0.001	0.63	0.60	0.67	<0.001	0.62	0.58	0.66
No		1				1		
Younger siblings								
Yes	<0.001	1.22	1.16	1.29	.655	0.99	0.92	1.05
No		1				1		
Breast- feeding								
<4 months (including none)	.594	0.98	0.92	1.05				
≧4 months		1						
Diagnosed asthma								
Yes	<0.001	3.12	2.88	3.39	<0.001	2.32	2.13	2.54
No		1				1		
Diagnosed eczema								
Yes	<0.001	2.28	2.16	2.42	<0.001	1.93	1.82	2.06
No		1				1		
Cats in the first year of life								
Yes	.002	1.31	1.10	1.56	.022	1.25	1.03	1.52
No		1				1		
Cats in the past 12 months								
Yes	.186	1.11	0.95	1.31				
No		1						
Dogs in the first year of life								
Yes	<0.001	1.22	1.12	1.31	.095	1.08	0.99	1.18
No		1				1		
Dogs in the past 12 months								
Yes	.561	1.03	0.94	1.12				
No		1						
Farm animals in the first year of life								
Yes	<0.001	1.38	1.16	1.63	.114	1.17	0.96	1.41
No		1				1		

C/S: Caesarean section; NSD: Normal spontaneous delivery; OR: Odd ratio. The multivariate analyses were adjusted for a post-hoc test by dividing the p -value of 0.05 with 10 (the amount of the significant factors in univariate analysis), that is, the corrected p -value is 0.005.

The prevalence of rhinitis in the past 12 months was 42.8% (10652/23630). The prevalence for males and females were 50% (6120/12236) and 39.8% (4532/11394) respectively. The prevalence of rhinitis in those who ever use antibiotics in the first year of life (n = 3566) was 58.1% (n = 2072). For children who are with bronchiolitis before the age of two, 2833 patients accounted for 61.1% of the total subjects answering "yes." There were 4468 patients (39.4%) had older siblings and 4075 (48.4%) had younger siblings. About 68.8% of first graders with diagnosed asthma (n = 3076) developed rhinitis in the past 12 months. And 58.8% (n = 4233) of first graders who answered "yes" to diagnosed eczema also developed rhinitis in the past 12 months. There were 275 patients (51.7%) had ever a cat at home and 1422 patients (49.4%) had ever a dog at home in the first year of life. For children who regularly exposed to farm animals in the first year of life, there were 288 patients accounting for 52.9% of the subjects answering “yes”.

Ten factors were statistically significant (p < 0.05) associated with rhinits and were included in the multi-factor logistic regression analysis: male gender, antibiotic use in the first year of life, bronchiolitis before the age of two, presence of older siblings, presence of younger siblings, diagnosed asthma, diagnosed eczema, cats in the first year of life, dogs in the first year of life, and farm animals in the first year of life. Of these gender (OR = 1.45, 95% CI = 1.37–1.54, p < 0.001), antibiotic use in the first year of life (OR = 1.34, 95% CI = 1.23–1.45, p < 0.001), bronchiolitis before age of two (OR = 1.68, 95% CI = 1.55–1.81, p < 0.001), diagnosed asthma (OR = 2.32, 95% CI = 2.13–2.54, p < 0.001), diagnosed eczema (OR = 1.93, 95% CI = 1.82–2.06, p < 0.001), and cats in the first year of life (OR = 1.25, 95% CI = 1.03–1.52) were positively associated with rhinitis, whereas the presence of older siblings were inversely related to rhinitis (OR = 0.62, 95% CI = 0.58–0.66, p < 0.001). The multivariate analyses were adjusted for a post-hoc test by dividing the p -value of 0.05 with the amount of the significant factors in univariate analysis, that is, the p-value of was 0.001. Only cat keeping is not significant after the adjustment. Table 4 indicates these risk factors had no collinearity. Interaction analysis (Table 5) shows some significant interactions with rhinitis symptoms, including bronchiolitis before the age of two * diagnosed asthma (p = .001), bronchiolitis before the age of two * diagnosed eczema (p = .011), bronchiolitis before the age of two * antibiotic use in the first year of life (p = .049), diagnosed eczema * older siblings * diagnosed asthma (p = .031), diagnosed eczema * older siblings * cats in the first year of life (p = .044), and diagnosed eczema * gender * antibiotic use in the first year of life (p = .009).

**Table 4:** The collinearity analysis for multivariate analysis of both males and females.

	Tolerance	VIF
Gender	.993	1.007
Antibiotic use in the first year of life	.897	1.114
Bronchiolitis before the age of two	.843	1.187
Older siblings	.797	1.254
Younger siblings	.805	1.243
Diagnosed asthma	.922	1.085
Diagnosed eczema	.955	1.047
Cats in the first year of life	.980	1.020
Dogs in the first year of life	.964	1.037
Farm animals in the first year of life	.981	1.019

**Table 5:** The interaction analysis for multivariate analysis of both males and females.

Source	df	Mean Square	F	p
Bronchiolitis before the age of two * Diagnosed asthma	1	2.304	10.284	.001
Bronchiolitis before the age of two * Diagnosed eczema	1	1.455	6.496	.011
Bronchiolitis before the age of two * Antibiotic use in the first year of life	1	.871	3.889	.049
Diagnosed eczema * Older siblings * Diagnosed asthma	1	1.038	4.633	.031
Diagnosed eczema * Older siblings * Cats in the first year of life	1	.913	4.076	.044
Diagnosed eczema * Gender * Antibiotic use in the first year of life	1	1.529	6.825	.009

**Fig 1:**
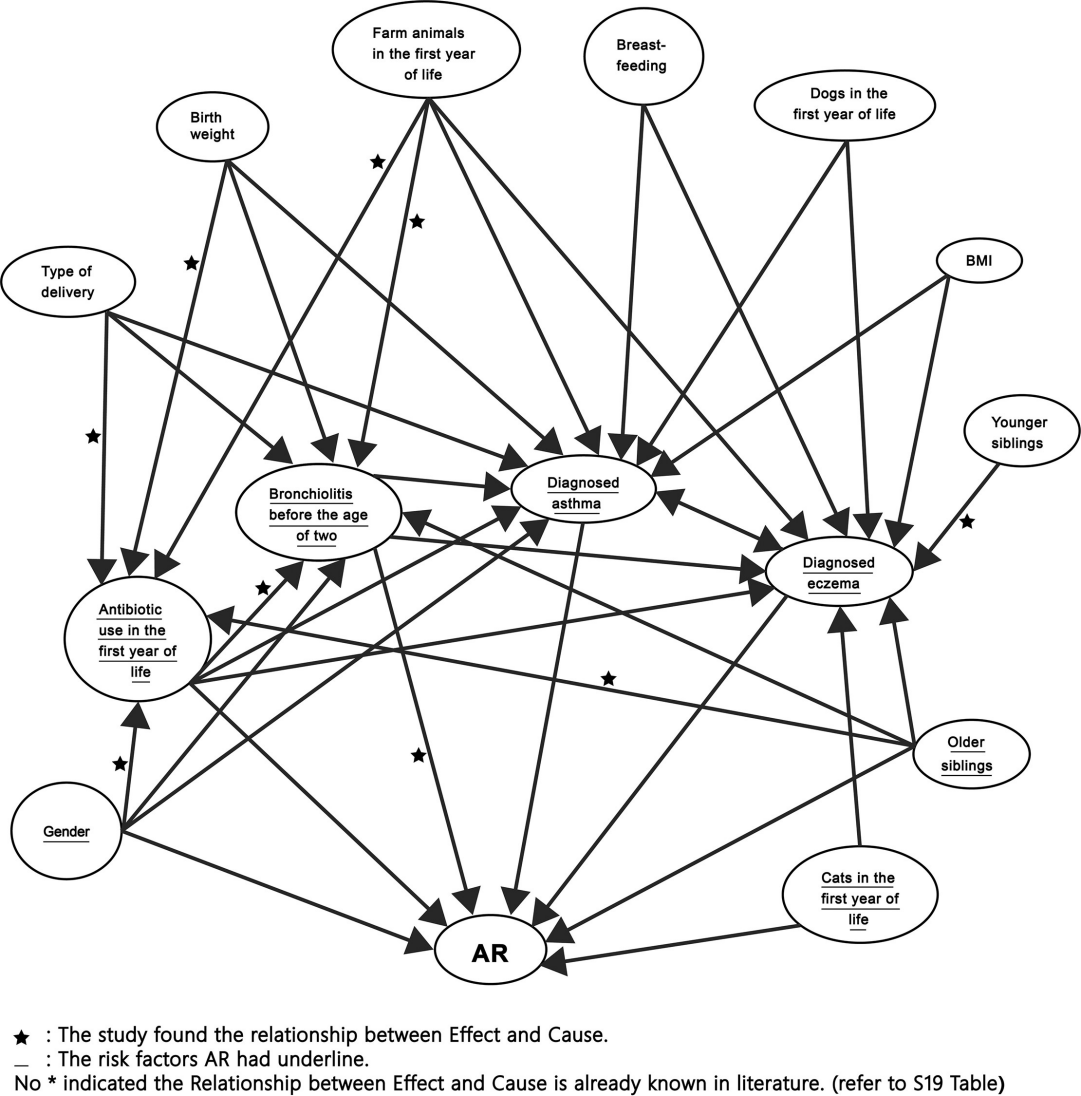
Directed acyclic graph of the rhinitis risk factors for both males and females.

The main risk factors of rhinitis and the main variables available indicate some significant interaction in Table 6. A directed acyclic graph (DAG) includes all strong evidences of association between effect and cause is in Fig 1.

**Table 6:** Lists of causal links and OR from logistic regression as indicated in the DAG for both males and females.

Effect	Cause	OR	p
Antibiotic use in the first year of life	Gender	1.255 (1.169–1.347)	<0.001
	Type of delivery	1.124 (1.045–1.209)	.002
	Birth weight	1.657 (1.462–1.877)	<0.001
	Older siblings	.914 (.852-.981)	.013
	Farm animals in the first year of life	1.421 (1.153–1.752)	.001
Bronchiolitis before the age of two	Gender	1.249 (1.172–1.331)	<0.001
	Type of delivery	1.145 (1.072–1.223)	<0.001
	Antibiotic use in the first year of life	5.532 (5.126–5.969)	<0.001
	Birth weight	1.391 (1.235–1.568)	<0.001
	Older siblings	1.205 (1.130–1.284)	<0.001
	Farm animals in the first year of life	1.705 (1.419–2.049)	<0.001
Diagnosed asthma	Gender	1.492 (1.383–1.609)	<0.001
	BMI	1.271 (1.165–1.386)	<0.001
	Type of delivery	1.143 (1.058–1.236)	.001
	Antibiotic use in the first year of life	2.062 (1.883–2.258)	<0.001
	Bronchiolitis before the age of two	4.375 (4.037–4.741)	<0.001
	Birth weight	1.429 (1.247–1.638)	<0.001
	Breast- feeding	.892 (.810-.981)	.019
	Diagnosed eczema	2.343 (2.169–2.532)	<0.001
	Dogs in the first year of life	1.210 (1.086–1.349)	.001
	Farm animals in the first year of life	1.491 (1.201–1.853)	<0.001
Diagnosed eczema	BMI	1.216 (1.139–1.299)	<0.001
	Antibiotic use in the first year of life	1.875 (1.742–2.017)	<0.001
	Bronchiolitis before the age of two	2.245 (2.101–2.399)	<0.001
	Older siblings	.763 (.721-.808)	<0.001
	Younger siblings	1.122 (1.059–1.188)	<0.001
	Breast- feeding	.886 (.825-.951)	.001
	Diagnosed asthma	2.343 (2.169–2.532)	<0.001
	Cats in the first year of life	1.209 (1.009–1.449)	.039
	Dogs in the first year of life	1.136 (1.045–1.233)	.003
	Farm animals in the first year of life	1.372 (1.152–1.633)	<0.001

OR, odd ratio.

The subgroups included males and females, only males, only females, only rhinitis, rhinitis & asthma, rhinitis & eczema, mild rhinitis, moderate rhinitis, severe rhinitis, and rhinitis ever. The severity of rhinitis was divided into groups as mild rhinitis, moderate rhinitis, and severe rhinitis. The relationship between the variables and rhinitis symptoms for subgroups is shown in Table 7.

**Table 7:** The relationship between the variables and rhinitis symptoms for subgroups.

	Males and females	males	females	only rhinitis	rhinitis & asthma	rhinitis & eczema	mild rhinitis	moderate rhinitis	severe rhinitis	rhinitis ever
Gender	1.45	n/a	n/a	1.46	1.50	1.43				1.46
BMI										
Type of delivery										
Antibiotic use in the first year of life	1.34	1.38	1.29	1.38	1.34	1.45	v	1.30	v	1.37
Bronchiolitis before the age of two	1.68	1.7	1.65	1.86	2.02	1.89	v	1.40	1.54	1.73
Birth weight										
Older siblings	0.62	0.60	0.64	0.62	0.60	0.61	1.19*			0.64
Younger siblings	v	v	v	v	v	v			v	
Breast- feeding										
Diagnosed asthma	2.32	2.38	2.26	n/a	n/a	n/a	1.88	2.27	3.24	2.43
Diagnosed eczema	1.93	1.90	1.97	n/a	n/a	n/a	1.54	2.00	2.65	1.94
Cats in the first year of life	1.25*		1.33*	1.34*	1.32*	v				1.24
Cats in the past 12 months										
Dogs in the first year of life	v	v	v	1.14	1.12*	1.12*				1.10
Dogs in the past 12 months										
Farm animals in the first year of life	v	v	v	1.31	v	1.25*				1.27

Results are presented for significant variables with odds ratios (OR) in the univariate analysis and the multivariable analysis with p < 0.05 and 95% confidence intervals (95% CI)**; v:** A significant factor in univariate analysis; n/a: Not Applicable

*: It is not a significant factor in multivariable analysis based on the p-value adjusting for a post-hoc test; The multivariate analyses were adjusted for a post-hoc test by dividing the p -value of 0.05 with the amount of the significant factors in univariate analysis, that is, the p-value of males and females, males, females, only rhinitis, rhinitis & asthma, rhinitis & eczema, mild rhinitis, moderate rhinitis, severe rhinitis, and rhinitis ever were 0.001, 0.0063, 0.0056, 0.0063, 0.0063, 0.0063, 0.01, 0.0125, 0.0083, 0.005, respectively. Please refer to the S1 Appendix.

The risk factors of males and females are the same as females except gender. The risk factors of males and females are one more risk factor, cats in the first year of life, than males. The differences among only rhinitis group, rhinitis & asthma group, and rhinitis & eczema group include cats in the first year of life and farm animals in the first year of life. The severity of rhinitis was divided into groups as mild rhinitis, moderate rhinitis, and severe rhinitis. The mild rhinitis group, the moderate rhinitis group, and the severe rhinitis group have the same risk factors, diagnosed asthma and diagnosed eczema. Other risk factors are different, including antibiotic use in the first year of life, bronchiolitis before the age of two, and older siblings. The first question of the ISSAC questionnaire for rhinitis, rhinitis ever, was also analyzed and its risk factors are 2 more than "rhinitis in the past 12 months", including dogs in the first year of life and farm animals in the first year of life.

## Discussion

This study found the prevalence of rhinitis was 42.8% in first graders in Taipei (northern Taiwan) in 2007. Around the world, the prevalence of rhinitis in children aged 6−7 years old is 18.7% in Poland [6], 29.2% in Turkey [7], 30.8% in Colombia [2], and 38.5% in Korea [8]. In central Taiwan, the prevalence of allergic rhinitis in children aged 6−15 years old in the year of 1987, 1994 and 2002 was 5.1% (n = 37,801), 12.46% (n = 75,960), and 27.59% (n = 11,580) respectively, showing that a 5.40-fold increase over a period of 14 years. The prevalence rate of allergic rhinitis was almost lower in the central area than the prevalence in northern and southern areas in Taiwan [9]. However, the prevalence of allergic rhinitis in Taiwan has increased markedly over the last decade and maintained high level compared with other regions.

Interaction analysis and the directed acyclic graph can better understand the relationship between rhinitis and variables, and the relationship between variables. The study analyzed ten subgroups, including males and females, only males, only females, only rhinitis, rhinitis & asthma, rhinitis & eczema, mild rhinitis, moderate rhinitis, severe rhinitis, and rhinitis ever. From these analyses, we can know the rhinitis risk factors of each subgroup and the influence of the severity of rhinitis.

In our study, rhinitis prevalence appeared higher in boys than girls. Similar findings have been reported in other studies in Taiwan [10–12]. In other country, Alm et al. conducted a prospective longitudinal study in 8,176 families randomly selected from a cohort found a rhinitis prevalence of 68% in boys and 32% in girls [13]. But some studies found very close rhinitis prevalence between the two genders. For example, the rhinitis prevalence of the 6–7 years old boys and girls in Istanbul were 50.7% and 49.3%, respectively [7]. Interestingly, the role of gender changes along with aging. In fact, the prevalence of atopic dermatitis before the age of 8 in females was lower than it in males, and the condition was reverse after the age. A similar trend was also reported in previous studies [14,15]. The reversal role of gender in rhinitis is similar to that seen in asthma [16–18], and sex hormones possibly play a role in the change [15,19]. In Taiwan, it is not yet to verify whether sex hormone is the factor of rhinitis.

Our study demonstrated that children who ever used antibiotics in the first year of life were 1.34-fold more likely to suffer from rhinitis than those who did not, which is consistent with some studies, including 1.8-fold [2], 1.75-fold [20], 1.51-fold [21], 1.60-fold [22], and 1.17-fold [23]. In Taiwan, government provides private medical institutions with reimbursement rates through the universal fee-for-service health care system. Patients often seek out for antibiotics treatment even the condition is not indicated. Physician who withholds antibiotics may lose the patients to other providers. Thus, doctors often feel pressured to prescribe antibiotics due to health care competition [24]. In five European countries, the antibiotic prescription rate on an infant varied among countries, ranging from 0.2 to 1.3 prescriptions per year [25]. Antibiotics may have an effect on the prevalence of allergic diseases through two pathways, including antibiotics may remove certain protective effect against allergies and the influence of antibiotics on the commensal bowel altering flora gut flora in atopic subjects [26]. Therefore, in case an infection is not bacterial, the antibiotic prescription rate on an infant must be reduced to prevent from rhinitis.

The most common cause of hospitalization in infancy is bronchiolitis, which is a burden for the child and family, and bears huge costs for the healthcare systems [27]. In Taiwan, 34.4% of children with bronchiolitis have asthma-like symptoms after entering kindergarten [28]. Although few studies put bronchiolitis into risk factors of rhinitis to evaluate, we considered it as a risk factor of rhinitis by clinical experience. The result showed that children with bronchiolitis before the age of two suffered from rhinitis more than those without bronchiolitis. Similar result was regarding the relation between bronchiolitis and asthma. For example, one study reported bronchiolitis had received particular attention as it had been suggested that an episode of bronchiolitis before the age of two causes subsequent lower respiratory morbidity or that bronchiolitis identifies those infants predisposed to develop asthma [29].

Results of some studies on analyzing the relation between siblings and rhinitis are consistent with ours, that having older siblings and larger sibling number is a protective effect in the development of rhinitis [30–32]. So far, there are no biological mechanisms known to cause the sibling effect. The sibling effect have been explained by the hygiene hypothesis and utero programming/endocrine explanatory models [33], but further studies are needed to test the models and the hypothesis. However, one study indicated only 3.9% of children had at least three siblings in Taiwan [29], and the lower birth rate will persist for a long time. Therefore, it is necessary to create a suitable environment for Taiwanese children, perhaps interactive activities with older children or relatives, to reduce rhinitis.

Children with diagnosed asthma or eczema were more likely to have rhinitis in our study, and it is consistent with other studies [1,2,8,14,34–36]. One study demonstrated a close relationship between rhinitis and asthma in children ever had asthma (OR = 2.32, 95% CI = 2.13–2.54, p < 0.001), as well as eczema (OR = 1.93, 95% CI = 1.82–2.06, p < 0.001) [35]. It confirms the concept that both asthma and eczema appear to be significant concomitant risk factors for rhinitis. Hwang and colleagues in 2010 analyzing the claims data of a nationally representative cohort of 997,729 enrollees from the National Health Insurance from 2000 to 2007 reported the prevalence of eczema, allergic rhinitis, and asthma in Taiwan. Overall, a total number of 262,665 (26.3%) patients were diagnosed with rhinitis, and 43.4% of them had concomitant eczema and/or asthma [36]. Therefore, children with diagnosed asthma or diagnosed eczema have higher chance to have rhinitis, which indicated the concept of allergy as a related condition mainly affecting the respiratory tract, the skin, and the nasal mucous. So, early management for eczema and asthma to prevent the other risk factors of rhinitis during the development of atopic march would be of critical importance.

After adjusting for Bonferroni correction, only cat keeping is not significant after the adjustment. Since most of the multivariate analysis of rhinitis literature used p<0.05 [2,5–7,20,21,30], this study was still based on p <0.05 to compare the difference. Some studies reported no evidences that keep with cats in the first year of life increased the risk of rhinitis [13,20] while others reported opposite findings. For example, a study reported that contact with pets at home increased the risk of rhinitis [35]. Interestingly, a study found that contact with 2 or more cats in the first year of life decreased the risk of atopy in childhood [37]. As observed in this study, about 51.7% of children keep with cats in the first year of life had rhinitis. Therefore, children who keep pets had a high prevalence of rhinitis in Taipei, and the underlying reasons for the contribution of number of cat exposure to rhinitis worth further investigation.

This study has several strengths, including a high response rate, a large sample size, multiple environmental factor interaction and the risk factors of AR to be explored in all districts in the Taipei metropolitan area. This study has potential limitations. First, we did not perform a doctor’s diagnosis to confirm the reported symptoms. Second, we should have the detailed items to explore the mechanism of the risk of rhinitis. Third, this study lacks allergy-test or lung-function confirmation of the disease to identify AR, asthma, and eczema. People with allergies can perform skin tests on specific allergens by scratch test or intradermal injection of allergens to distinguish internal and external causes and therefore to develop treatment strategies. However, this study is to identify relevant risk factors, so our results should be valid. Hence, we can use this result to explore the more detailed risk factors of rhinitis by designing the appropriate questionnaire.

## Conclusion

Our study verified personal and environmental risk factors of rhinitis in children in Taiwan for many subgroups. The prevalence of rhinitis remained high among first graders in Taipei. We recommend an evidence-based health education for parents and main caregivers to teach the different living activities (perhaps interactive activities with older children or relatives) to reduce the prevalence of rhinitis, the alertness and treatment on common comorbid disease (i.e. asthma and eczema), proper use of antibiotics in the first year of life, and no cats in the house in the first year. Moreover, from the subgroup analysis, we can take preventive measures for the different risk factors of rhinitis and the severity of rhinitis in each subgroup. Further studies of the biological mechanisms of the risk factors of AR are warranted.

## Supporting information

S1 Appendix (DOCX)
