# Supplementary material for: Risk factor analysis of allergic rhinitis in 6–8 year-old children in Taipei
Source: PLoS One. 2021 Apr 2;16(4):e0249572. doi: 10.1371/journal.pone.0249572 (PMC8018651; doi:10.1371/journal.pone.0249572)
Supplement: S1 Appendix — (DOCX) [file pone.0249572.s001.docx]

## Supporting information

## S1 Table. The relationship between the personal factors and rhinitis symptoms for males in question 2.

|  | **No rhinitis**  **(*n* = 6116)** |  | **rhinitis**  **(n = 6120)** |  |  |  | **Univariate** | | |  |  | |  | **Multivariate** | | | |
| --- | --- | --- | --- | --- | --- | --- | --- | --- | --- | --- | --- | --- | --- | --- | --- | --- | --- |
| **Variable** | ***n (%)*** |  | ***N*** |  | ***P*** | | ***OR*** | ***lower*** | ***upper*** |  | ***P*** | ***OR*** | | | ***lower*** | ***upper*** |  |
| BMI |  |  |  |  |  | |  |  |  |  |  |  | | |  |  |  |
| Overweight/ obesity | 1597 (49.1) |  | 1657 (50.9) |  | .573 | | 0.98 | 0.90 | 1.06 |  |  |  | | |  |  |  |
| Underweight/ normal | 3413 (48.5) |  | 3627 (51.5) |  |  | | 1 |  |  |  |  |  | | |  |  |  |
| Type of delivery |  |  |  |  |  | |  |  |  |  |  |  | | |  |  |  |
| C/S | 2186 (49.7) |  | 2209 (50.3) |  | .798 | | 1.01 | 0.94 | 1.09 |  |  |  | | |  |  |  |
| NSD | 3816 (50.0) |  | 3819 (50.0) |  |  | | 1 |  |  |  |  |  | | |  |  |  |
| Antibiotic use in the first year of life |  |  |  |  |  | |  |  |  |  |  |  | | |  |  |  |
| Yes | 738 (36.8) |  | 1270 (63.2) |  | <0.001 | | 1.92 | 1.74 | 2.12 |  | <0.001 | 1.38 | | | 1.24 | 1.55 |  |
| No (including unknown) | 5223 (52.7) |  | 4683 (47.3) |  |  | | 1 |  |  |  |  | 1 | | |  |  |  |
| Bronchiolitis before the age of two |  |  |  |  |  | |  |  |  |  |  |  | | |  |  |  |
| Yes | 738 (36.8) |  | 1270 (63.2) |  | <0.001 | | 2.31 | 2.11 | 2.53 |  | <0.001 | 1.70 | | | 1.53 | 1.89 |  |
| No (including unknown) | 5223 (52.7) |  | 4683 (47.3) |  |  | | 1 |  |  |  |  | 1 | | |  |  |  |
| Birth weight |  |  |  |  |  | |  |  |  |  |  |  | | |  |  |  |
| Underweight (<2500 g) | 5126 (49.0) |  | 5336 (51.0) |  | .654 | | 1.04 | 0.89 | 1.21 |  |  |  | | |  |  |  |
| Normal (≧2500 g) | 5126 (49.0) |  | 5336 (51.0) |  |  | | 1 |  |  |  |  |  | | |  |  |  |
| Older siblings |  |  |  |  |  | |  |  |  |  |  |  | | |  |  |  |
| Yes | 3316 (56.1) |  | 2594 (43.9) |  | <0.001 | | 0.61 | 0.57 | 0.66 |  | <0.001 | 0.60 | | | 0.55 | 0.65 |  |
| No | 2724 (44.0) |  | 3467 (56.0) |  |  | | 1 |  |  |  |  | 1 | | |  |  |  |
| Younger siblings |  |  |  |  |  | |  |  |  |  |  |  | | |  |  |  |
| Yes | 1907 (45.9) |  | 2245 (54.1) |  | <0.001 | | 1.28 | 1.18 | 1.37 |  | .789 | 0.99 | | | 0.90 | 1.08 |  |
| No | 4133 (52.0) |  | 3816 (48.0) |  |  | | 1 |  |  |  |  | 1 | | |  |  |  |
| Breast- feeding |  |  |  |  |  | |  |  |  |  |  |  | | |  |  |  |
| <4 months (including none) | 4924 (49.9) |  | 4944 (50.1) |  | .795 | | 1.01 | 0.92 | 1.11 |  |  |  | | |  |  |  |
| ≧4 months | 1096 (50.2) |  | 1087 (49.8) |  |  | | 1 |  |  |  |  |  | | |  |  |  |
| Diagnosed asthma |  |  |  |  |  | |  |  |  |  |  |  | | |  |  |  |
| Yes | 514 (27.7) |  | 1340 (72.3) |  | <0.001 | | 3.07 | 2.75 | 3.42 |  | <0.001 | 2.38 | | | 2.11 | 2.68 |  |
| No | 5537 (54.0) |  | 4709 (46.0) |  |  | | 1 |  |  |  |  | 1 | | |  |  |  |
| Diagnosed eczema |  |  |  |  |  | |  |  |  |  |  |  | | |  |  |  |
| Yes | 1366 (36.2) |  | 2411 (63.8) |  | <0.001 | | 2.33 | 2.15 | 2.52 |  | <0.001 | 1.90 | | | 1.74 | 2.07 |  |
| No | 4539 (56.9) |  | 3443 (43.1) |  |  | | 1 |  |  |  |  | 1 | | |  |  |  |
| Cats in the first year of life |  |  |  |  |  | |  |  |  |  |  |  | | |  |  |  |
| Yes | 125 (47.0) |  | 141 (53.0) |  | .324 | | 1.13 | 0.89 | 1.44 |  |  |  | | |  |  |  |
| No | 5944 (50.1) |  | 5932 (49.9) |  |  | | 1 |  |  |  |  |  | | |  |  |  |
| Cats in the past 12 months |  |  |  |  |  | |  |  |  |  |  |  | | |  |  |  |
| Yes | 146 (47.6) |  | 161 (52.4) |  | .394 | | 1.10 | 0.88 | 1.38 |  |  |  | | |  |  |  |
| No | 5919 (50.0) |  | 5914 (50.0) |  |  | | 1 |  |  |  |  |  | | |  |  |  |
| Dogs in the first year of life |  |  |  |  |  | |  |  |  |  |  |  | | |  |  |  |
| Yes | 710 (46.8) |  | 807 (53.2) |  | .009 | | 1.15 | 1.04 | 1.28 |  | .382 | 1.05 | | | 0.94 | 1.19 |  |
| No | 5352 (50.4) |  | 5273 (49.6) |  |  | | 1 |  |  |  |  | 1 | | |  |  |  |
| Dogs in the past 12 months |  |  |  |  |  | |  |  |  |  |  |  | | |  |  |  |
| Yes | 615 (49.4) |  | 631 (50.6) |  | .661 | | 1.03 | 0.91 | 1.15 |  |  |  | | |  |  |  |
| No | 5452 (50.0) |  | 5449 (50.0) |  |  | | 1 |  |  |  |  |  | | |  |  |  |
| Farm animals in the first year of life |  |  |  |  |  | |  |  |  |  |  |  | | |  |  |  |
| Yes | 126 (43.4) |  | 164 (56.6) |  | .025 | | 1.31 | 1.03 | 1.65 |  | .521 | 1.09 | | | 0.84 | 1.42 |  |
| No | 5941 (50.1) |  | 5910 (49.9) |  |  | | 1 |  |  |  |  | 1 | | |  |  |  |

C/S: Caesarean section; NSD: Normal spontaneous delivery

The p < 0.05 is in univariate analysis.

The multivariate analyses were adjusted for a post-hoc test by dividing the p -value of 0.05 with 8 (the amount of the significant factors in univariate analysis), that is, the corrected p -value is about 0.0063.

OR, odd ratio

## S2 Table. The collinearity analysis for S1 Table.

|  | **Tolerance** | **VIF** |
| --- | --- | --- |
| Antibiotic use in the first year of life | .892 | 1.121 |
| Bronchiolitis before the age of two | .831 | 1.204 |
| Older siblings | .781 | 1.280 |
| Younger siblings | .789 | 1.268 |
| Diagnosed asthma | .918 | 1.090 |
| Diagnosed eczema | .948 | 1.054 |
| Dogs in the first year of life | .980 | 1.020 |
| Farm animals in the first year of life | .982 | 1.018 |

## S3 Table. The relationship between the personal factors and rhinitis symptoms for females in question 1 & 2.

|  | **No rhinitis**  **(*n* = 6862)** |  | **rhinitis**  **(n = 4532)** |  |  |  | **Univariate** | | |  |  | |  | **Multivariate** | | | |
| --- | --- | --- | --- | --- | --- | --- | --- | --- | --- | --- | --- | --- | --- | --- | --- | --- | --- |
| **Variable** | ***n (%)*** |  | ***N*** |  | ***P*** | | ***OR*** | ***lower*** | ***upper*** |  | ***P*** | ***OR*** | | | ***lower*** | ***upper*** |  |
| BMI |  |  |  |  |  | |  |  |  |  |  |  | | |  |  |  |
| Overweight/ obesity | 1353 (60.1) |  | 898 (39.9) |  | .251 | | 0.95 | 0.86 | 1.04 |  |  |  | | |  |  |  |
| Underweight/ normal | 4279 (58.7) |  | 3005 (41.3) |  |  | | 1 |  |  |  |  |  | | |  |  |  |
| Type of delivery |  |  |  |  |  | |  |  |  |  |  |  | | |  |  |  |
| C/S | 2297 (59.3) |  | 1578 (40.7) |  | .182 | | 1.06 | 0.97 | 1.14 |  |  |  | | |  |  |  |
| NSD | 4425 (60.6) |  | 2880 (39.4) |  |  | | 1 |  |  |  |  |  | | |  |  |  |
| Antibiotic use in the first year of life |  |  |  |  |  | |  |  |  |  |  |  | | |  |  |  |
| Yes | 756 (48.5) |  | 802 (51.5) |  | <0.001 | | 1.72 | 1.55 | 1.92 |  | <0.001 | 1.29 | | | 1.14 | 1.45 |  |
| No (including unknown) | 5915 (61.9) |  | 3639 (38.1) |  |  | | 1 |  |  |  |  | 1 | | |  |  |  |
| Bronchiolitis before the age of two |  |  |  |  |  | |  |  |  |  |  |  | | |  |  |  |
| Yes | 914 (44.9) |  | 1120 (55.1) |  | <0.001 | | 2.14 | 1.94 | 2.36 |  | <0.001 | 1.65 | | | 1.48 | 1.85 |  |
| No (including unknown) | 5765 (63.6) |  | 3303 (36.4) |  |  | | 1 |  |  |  |  | 1 | | |  |  |  |
| Birth weight |  |  |  |  |  | |  |  |  |  |  |  | | |  |  |  |
| Underweight (<2500 g) | 474 (57.5) |  | 350 (42.5) |  | .253 | | 1.09 | 0.94 | 1.26 |  |  |  | | |  |  |  |
| Normal (≧2500 g) | 5642 (59.6) |  | 3830 (40.4) |  |  | | 1 |  |  |  |  |  | | |  |  |  |
| Older siblings |  |  |  |  |  | |  |  |  |  |  |  | | |  |  |  |
| Yes | 3562 (65.5) |  | 1874 (34.5) |  | <0.001 | | 0.64 | 0.59 | 0.69 |  | <0.001 | 0.64 | | | 0.59 | 0.71 |  |
| No | 3204 (55.0) |  | 2626 (45.0) |  |  | | 1 |  |  |  |  | 1 | | |  |  |  |
| Younger siblings |  |  |  |  |  | |  |  |  |  |  |  | | |  |  |  |
| Yes | 2438 (57.1) |  | 1830 (42.9) |  | <0.001 | | 1.22 | 1.13 | 1.31 |  | .695 | 0.98 | | | 0.90 | 1.08 |  |
| No | 4328 (61.8) |  | 2670 (38.2) |  |  | | 1 |  |  |  |  | 1 | | |  |  |  |
| Breast- feeding |  |  |  |  |  | |  |  |  |  |  |  | | |  |  |  |
| <4 months (including none) | 5572 (60.4) |  | 3654 (39.6) |  | .331 | | 1.22 | 1.13 | 1.31 |  |  |  | | |  |  |  |
| ≧4 months | 1185 (59.2) |  | 816 (40.8) |  |  | | 1 |  |  |  |  |  | | |  |  |  |
| Diagnosed asthma |  |  |  |  |  | |  |  |  |  |  |  | | |  |  |  |
| Yes | 445 (36.4) |  | 777 (63.6) |  | <0.001 | | 3.00 | 2.65 | 3.40 |  | <0.001 | 2.26 | | | 1.97 | 2.58 |  |
| No | 6365 (63.2) |  | 3703 (36.8) |  |  | | 1 |  |  |  |  | 1 | | |  |  |  |
| Diagnosed eczema |  |  |  |  |  | |  |  |  |  |  |  | | |  |  |  |
| Yes | 1604 (46.8) |  | 1822 (53.2) |  | <0.001 | | 2.26 | 2.08 | 2.45 |  | <0.001 | 1.97 | | | 1.80 | 2.15 |  |
| No | 5043 (66.5) |  | 2535 (33.5) |  |  | | 1 |  |  |  |  | 1 | | |  |  |  |
| Cats in the first year of life |  |  |  |  |  | |  |  |  |  |  |  | | |  |  |  |
| Yes | 132 (49.6) |  | 134 (50.4) |  | <0.001 | | 1.55 | 1.22 | 1.98 |  | .037 | 1.33 | | | 1.02 | 1.75 |  |
| No | 6684 (60.5) |  | 4371 (39.5) |  |  | | 1 |  |  |  |  | 1 | | |  |  |  |
| Cats in the past 12 months |  |  |  |  |  | |  |  |  |  |  |  | | |  |  |  |
| Yes | 177 (56.9) |  | 134 (43.1) |  | .237 | | 1.15 | 0.91 | 1.44 |  |  |  | | |  |  |  |
| No | 6630 (60.2) |  | 4375 (39.8) |  |  | | 1 |  |  |  |  |  | | |  |  |  |
| Dogs in the first year of life |  |  |  |  |  | |  |  |  |  |  |  | | |  |  |  |
| Yes | 747 (54.8) |  | 615 (45.2) |  | <0.001 | | 1.28 | 1.14 | 1.44 |  | .111 | 1.11 | | | 0.98 | 1.26 |  |
| No | 6061 (60.9) |  | 3892 (39.1) |  |  | | 1 |  |  |  |  | 1 | | |  |  |  |
| Dogs in the past 12 months |  |  |  |  |  | |  |  |  |  |  |  | | |  |  |  |
| Yes | 703 (59.6) |  | 477 (40.4) |  | .650 | | 1.03 | 0.91 | 1.16 |  |  |  | | |  |  |  |
| No | 6103 (60.3) |  | 4025 (39.7) |  |  | | 1 |  |  |  |  |  | | |  |  |  |
| Farm animals in the first year of life |  |  |  |  |  | |  |  |  |  |  |  | | |  |  |  |
| Yes | 130 (51.2) |  | 124 (48.8) |  | .003 | | 1.45 | 1.13 | 1.86 |  | .092 | 1.27 | | | 0.96 | 1.68 |  |
| No | 6673 (60.3) |  | 4390 (39.7) |  |  | | 1 |  |  |  |  | 1 | | |  |  |  |

C/S: Caesarean section; NSD: Normal spontaneous delivery

The p < 0.05 is in univariate analysis.

The multivariate analyses were adjusted for a post-hoc test by dividing the p -value of 0.05 with 9 (the amount of the significant factors in univariate analysis), that is, the corrected p -value is about 0.0056.

OR, odd ratio

## S4 Table. The collinearity analysis for S3 Table.

|  | **Tolerance** | **VIF** |
| --- | --- | --- |
| Antibiotic use in the first year of life | .906 | 1.103 |
| Bronchiolitis before the age of two | .860 | 1.163 |
| Older siblings | .813 | 1.230 |
| Younger siblings | .823 | 1.216 |
| Diagnosed asthma | .934 | 1.070 |
| Diagnosed eczema | .961 | 1.040 |
| Cats in the first year of life | .968 | 1.033 |
| Dogs in the first year of life | .957 | 1.045 |
| Farm animals in the first year of life | .977 | 1.024 |

## S5 Table. The relationship between the personal factors and rhinitis symptoms for only rhinitis in question 1 & 2.

|  | **No rhinitis**  **(*n* = 9387)** |  | **rhinitis**  **(n = 5403)** |  |  |  | **Univariate** | | |  |  | |  | **Multivariate** | | | |
| --- | --- | --- | --- | --- | --- | --- | --- | --- | --- | --- | --- | --- | --- | --- | --- | --- | --- |
| **Variable** | ***n (%)*** |  | ***N*** |  | ***P*** | | ***OR*** | ***lower*** | ***upper*** |  | ***P*** | ***OR*** | | | ***lower*** | ***upper*** |  |
| Gender |  |  |  |  |  | |  |  |  |  |  |  | | |  |  |  |
| Male | 4414 (59.1) |  | 3058 (40.9) |  | <0.001 | | 1.47 | 1.37 | 1.57 |  | <0.001 | 1.46 | | | 1.36 | 1.56 |  |
| Female | 4973 (68.0) |  | 2345 (32.0) |  |  | | 1 |  |  |  |  | 1 | | |  |  |  |
| BMI |  |  |  |  |  | |  |  |  |  |  |  | | |  |  |  |
| Overweight/ obesity | 2026 (63.7) |  | 1156 (36.3) |  | .084 | | 0.93 | 0.85 | 1.01 |  |  |  | | |  |  |  |
| Underweight/ normal | 5590 (61.9) |  | 3434 (38.1) |  |  | | 1 |  |  |  |  |  | | |  |  |  |
| Type of delivery |  |  |  |  |  | |  |  |  |  |  |  | | |  |  |  |
| C/S | 3242 (63.3) |  | 1879 (36.7) |  | .857 | | 1.01 | 0.94 | 1.08 |  |  |  | | |  |  |  |
| NSD | 5948 (63.5) |  | 3425 (36.5) |  |  | | 1 |  |  |  |  |  | | |  |  |  |
| Antibiotic use in the first year of life |  |  |  |  |  | |  |  |  |  |  |  | | |  |  |  |
| Yes | 893 (52.6) |  | 805 (47.4) |  | <0.001 | | 1.67 | 1.50 | 1.84 |  | <0.001 | 1.38 | | | 1.23 | 1.53 |  |
| No (including unknown) | 8226 (64.9) |  | 4452 (35.1) |  |  | | 1 |  |  |  |  | 1 | | |  |  |  |
| Bronchiolitis before the age of two (1094, 4.4%) |  |  |  |  |  | |  |  |  |  |  |  | | |  |  |  |
| Yes | 946 (49.3) |  | 974 (50.7) |  | <0.001 | | 1.97 | 1.79 | 2.17 |  | <0.001 | 1.86 | | | 1.67 | 2.06 |  |
| No (including unknown) | 8203 (65.7) |  | 4283 (34.3) |  |  | | 1 |  |  |  |  | 1 | | |  |  |  |
| Birth weight |  |  |  |  |  | |  |  |  |  |  |  | | |  |  |  |
| Underweight (<2500 g) | 569 (63.6) |  | 326 (36.4) |  | .528 | | 0.96 | 0.83 | 1.10 |  |  |  | | |  |  |  |
| Normal (≧2500 g) | 7718 (62.5) |  | 4627 (37.5) |  |  | | 1 |  |  |  |  |  | | |  |  |  |
| Older siblings |  |  |  |  |  | |  |  |  |  |  |  | | |  |  |  |
| Yes | 5060 (68.5) |  | 2323 (31.5) |  | <0.001 | | 0.64 | 0.60 | 0.68 |  | <0.001 | 0.62 | | | 0.57 | 0.67 |  |
| No | 4187 (58.1) |  | 3018 (41.9) |  |  | | 1 |  |  |  |  | 1 | | |  |  |  |
| Younger siblings |  |  |  |  |  | |  |  |  |  |  |  | | |  |  |  |
| Yes | 3118 (60.5) |  | 2032 (39.5) |  | <0.001 | | 1.21 | 1.13 | 1.29 |  | .643 | 0.98 | | | 0.90 | 1.06 |  |
| No | 6129 (64.9) |  | 3309 (35.1) |  |  | | 1 |  |  |  |  | 1 | | |  |  |  |
| Breast- feeding |  |  |  |  |  | |  |  |  |  |  |  | | |  |  |  |
| <4 months (including none) | 7605 (63.2) |  | 4427 (36.8) |  | .238 | | 1.06 | 0.96 | 1.15 |  |  |  | | |  |  |  |
| ≧4 months | 1621 (64.5) |  | 894 (35.5) |  |  | | 1 |  |  |  |  |  | | |  |  |  |
| Cats in the first year of life |  |  |  |  |  | |  |  |  |  |  |  | | |  |  |  |
| Yes | 170 (55.2) |  | 138 (44.8) |  | .002 | | 1.42 | 1.13 | 1.79 |  | .017 | 1.34 | | | 1.05 | 1.71 |  |
| No | 9138 (63.7) |  | 5215 (36.3) |  |  | | 1 |  |  |  |  | 1 | | |  |  |  |
| Cats in the past 12 months |  |  |  |  |  | |  |  |  |  |  |  | | |  |  |  |
| Yes | 237 (59.7) |  | 160 (40.3) |  | .117 | | 1.18 | 0.96 | 1.44 |  |  |  | | |  |  |  |
| No | 9059 (63.5) |  | 5197 (36.5) |  |  | | 1 |  |  |  |  |  | | |  |  |  |
| Dogs in the first year of life |  |  |  |  |  | |  |  |  |  |  |  | | |  |  |  |
| Yes | 998 (59.1) |  | 692 (40.9) |  | <0.001 | | 1.23 | 1.11 | 1.37 |  | .020 | 1.14 | | | 1.02 | 1.27 |  |
| No | 8299 (64.0) |  | 4665 (36.0) |  |  | | 1 |  |  |  |  | 1 | | |  |  |  |
| Dogs in the past 12 months |  |  |  |  |  | |  |  |  |  |  |  | | |  |  |  |
| Yes | 943 (62.3) |  | 571 (37.7) |  | .318 | | 1.06 | 0.95 | 1.18 |  |  |  | | |  |  |  |
| No | 8357 (63.6) |  | 4785 (36.4) |  |  | | 1 |  |  |  |  |  | | |  |  |  |
| Farm animals in the first year of life |  |  |  |  |  | |  |  |  |  |  |  | | |  |  |  |
| Yes | 166 (55.5) |  | 133 (44.5) |  | .004 | | 1.40 | 1.11 | 1.77 |  | .031 | 1.31 | | | 1.02 | 1.67 |  |
| No | 9133 (63.6) |  | 5221 (36.4) |  |  | | 1 |  |  |  |  | 1 | | |  |  |  |

C/S: Caesarean section; NSD: Normal spontaneous delivery

The p < 0.05 is in univariate analysis.

The multivariate analyses were adjusted for a post-hoc test by dividing the p -value of 0.05 with 8 (the amount of the significant factors in univariate analysis), that is, the corrected p -value is about 0.0063.

## S6 Table. The collinearity analysis for S5 Table.

|  | **Tolerance** | **VIF** |
| --- | --- | --- |
| Gender | .998 | 1.002 |
| Antibiotic use in the first year of life | .927 | 1.078 |
| Bronchiolitis before the age of two | .926 | 1.080 |
| Older siblings | .808 | 1.237 |
| Younger siblings | .812 | 1.231 |
| Cats in the first year of life | .981 | 1.020 |
| Dogs in the first year of life | .968 | 1.033 |
| Farm animals in the first year of life | .985 | 1.016 |

## S7 Table. The relationship between the personal factors and rhinitis symptoms for rhinitis & asthma in question 1 & 2.

|  | **No rhinitis**  **(*n* = 10008)** |  | **rhinitis**  **(n = 6419)** |  |  |  | **Univariate** | | |  |  | |  | **Multivariate** | | | |
| --- | --- | --- | --- | --- | --- | --- | --- | --- | --- | --- | --- | --- | --- | --- | --- | --- | --- |
| **Variable** | ***n (%)*** |  | ***N*** |  | ***P*** | | ***OR*** | ***lower*** | ***upper*** |  | ***P*** | ***OR*** | | | ***lower*** | ***upper*** |  |
| Gender |  |  |  |  |  | |  |  |  |  |  |  | | |  |  |  |
| Male | 4750 (56.2) |  | 3709 (43.8) |  | <0.001 | | 1.52 | 1.42 | 1.61 |  | <0.001 | 1.50 | | | 1.40 | 1.60 |  |
| Female | 5258 (66.0) |  | 2710 (34.0) |  |  | | 1 |  |  |  |  | 1 | | |  |  |  |
| BMI |  |  |  |  |  | |  |  |  |  |  |  | | |  |  |  |
| Overweight/ obesity | 2178 (60.5) |  | 1422 (39.5) |  | .335 | | 0.96 | 0.89 | 1.04 |  |  |  | | |  |  |  |
| Underweight/ normal | 5951 (59.6) |  | 4037 (40.4) |  |  | | 1 |  |  |  |  |  | | |  |  |  |
| Type of delivery |  |  |  |  |  | |  |  |  |  |  |  | | |  |  |  |
| C/S | 3466 (60.7) |  | 2246 (39.3) |  | .743 | | 1.01 | 0.95 | 1.08 |  |  |  | | |  |  |  |
| NSD | 6335 (60.9) |  | 4060 (39.1) |  |  | | 1 |  |  |  |  |  | | |  |  |  |
| Antibiotic use in the first year of life |  |  |  |  |  | |  |  |  |  |  |  | | |  |  |  |
| Yes | 1017 (49.6) |  | 1032 (50.4) |  | <0.001 | | 1.70 | 1.55 | 1.86 |  | <0.001 | 1.34 | | | 1.21 | 1.48 |  |
| No (including unknown) | 8711 (62.6) |  | 5209 (37.4) |  |  | | 1 |  |  |  |  | 1 | | |  |  |  |
| Bronchiolitis before the age of two |  |  |  |  |  | |  |  |  |  |  |  | | |  |  |  |
| Yes | 1161 (45.4) |  | 1396 (54.6) |  | <0.001 | | 2.13 | 1.96 | 2.32 |  | <0.001 | 2.02 | | | 1.85 | 2.22 |  |
| No (including unknown) | 8586 (63.9) |  | 4844 (36.1) |  |  | | 1 |  |  |  |  | 1 | | |  |  |  |
| Birth weight |  |  |  |  |  | |  |  |  |  |  |  | | |  |  |  |
| Underweight (<2500 g) | 623 (59.2) |  | 429 (40.8) |  | .619 | | 1.03 | 0.91 | 1.17 |  |  |  | | |  |  |  |
| Normal (≧2500 g) | 8223 (60.0) |  | 5482 (40.0) |  |  | | 1 |  |  |  |  |  | | |  |  |  |
| Older siblings |  |  |  |  |  | |  |  |  |  |  |  | | |  |  |  |
| Yes | 5434 (66.2) |  | 2776 (33.8) |  | <0.001 | | 0.63 | 0.59 | 0.67 |  | <0.001 | 0.60 | | | 0.56 | 0.65 |  |
| No | 4430 (55.4) |  | 3573 (44.6) |  |  | | 1 |  |  |  |  | 1 | | |  |  |  |
| Younger siblings |  |  |  |  |  | |  |  |  |  |  |  | | |  |  |  |
| Yes | 3311 (58.0) |  | 2402 (42.0) |  | <0.001 | | 1.20 | 1.13 | 1.29 |  | .506 | 0.97 | | | 0.90 | 1.05 |  |
| No | 6553 (62.4) |  | 3947 (37.6) |  |  | | 1 |  |  |  |  | 1 | | |  |  |  |
| Breast- feeding |  |  |  |  |  | |  |  |  |  |  |  | | |  |  |  |
| <4 months (including none) | 8097 (60.7) |  | 5239 (39.3) |  | .307 | | 1.04 | 0.96 | 1.14 |  |  |  | | |  |  |  |
| ≧4 months | 1737 (61.7) |  | 1076 (38.3) |  |  | | 1 |  |  |  |  |  | | |  |  |  |
| Cats in the first year of life |  |  |  |  |  | |  |  |  |  |  |  | | |  |  |  |
| Yes | 186 (53.3) |  | 163 (46.7) |  | .003 | | 1.38 | 1.11 | 1.70 |  | .017 | 1.32 | | | 1.05 | 1.65 |  |
| No | 9740 (61.1) |  | 6200 (38.9) |  |  | | 1 |  |  |  |  | 1 | | |  |  |  |
| Cats in the past 12 months |  |  |  |  |  | |  |  |  |  |  |  | | |  |  |  |
| Yes | 253 (57.5) |  | 187 (42.5) |  | .139 | | 1.16 | 0.95 | 1.40 |  |  |  | | |  |  |  |
| No | 9662 (61.0) |  | 6179 (39.0) |  |  | | 1 |  |  |  |  |  | | |  |  |  |
| Dogs in the first year of life |  |  |  |  |  | |  |  |  |  |  |  | | |  |  |  |
| Yes | 1085 (56.4) |  | 838 (43.6) |  | <0.001 | | 1.23 | 1.12 | 1.36 |  | .027 | 1.12 | | | 1.01 | 1.25 |  |
| No | 8829 (61.5) |  | 5529 (38.5) |  |  | | 1 |  |  |  |  | 1 | | |  |  |  |
| Dogs in the past 12 months |  |  |  |  |  | |  |  |  |  |  |  | | |  |  |  |
| Yes | 1010 (59.4) |  | 690 (40.6) |  | .183 | | 1.07 | 0.97 | 1.19 |  |  |  | | |  |  |  |
| No | 8907 (61.1) |  | 5676 (38.9) |  |  | | 1 |  |  |  |  |  | | |  |  |  |
| Farm animals in the first year of life |  |  |  |  |  | |  |  |  |  |  |  | | |  |  |  |
| Yes | 186 (54.4) |  | 156 (45.6) |  | .013 | | 1.31 | 1.06 | 1.63 |  | .095 | 1.22 | | | 0.97 | 1.53 |  |
| No | 9732 (61.1) |  | 6208 (38.9) |  |  | | 1 |  |  |  |  | 1 | | |  |  |  |

C/S: Caesarean section; NSD: Normal spontaneous delivery

The p < 0.05 is in univariate analysis.

The multivariate analyses were adjusted for a post-hoc test by dividing the p -value of 0.05 with 8 (the amount of the significant factors in univariate analysis), that is, the corrected p -value is about 0.0063.

## S8 Table. The collinearity analysis for S7 Table.

|  | **Tolerance** | **VIF** |
| --- | --- | --- |
| Gender | .994 | 1.006 |
| Antibiotic use in the first year of life | .914 | 1.094 |
| Bronchiolitis before the age of two | .872 | 1.147 |
| Older siblings | .804 | 1.244 |
| Younger siblings | .809 | 1.237 |
| Cats in the first year of life | .944 | 1.060 |
| Dogs in the first year of life | .981 | 1.020 |
| Farm animals in the first year of life | .966 | 1.036 |

## S9 Table. The relationship between the personal factors and rhinitis symptoms for rhinitis & eczema in question 1 & 2.

|  | **No rhinitis**  **(*n* = 12019)** |  | **rhinitis**  **(n = 8535)** |  |  |  | **Univariate** | | |  |  | |  | **Multivariate** | | | |
| --- | --- | --- | --- | --- | --- | --- | --- | --- | --- | --- | --- | --- | --- | --- | --- | --- | --- |
| **Variable** | ***n (%)*** |  | ***N*** |  | ***P*** | | ***OR*** | ***lower*** | ***upper*** |  | ***P*** | ***OR*** | | | ***lower*** | ***upper*** |  |
| Gender |  |  |  |  |  | |  |  |  |  |  |  | | |  |  |  |
| Male | 5602 (54.0) |  | 4780 (46.0) |  | <0.001 | | 1.46 | 1.38 | 1.54 |  | <0.001 | 1.43 | | | 1.35 | 1.51 |  |
| Female | 6417 (63.1) |  | 3755 (36.9) |  |  | | 1 |  |  |  |  | 1 | | |  |  |  |
| BMI |  |  |  |  |  | |  |  |  |  |  |  | | |  |  |  |
| Overweight/ obesity | 2691 (57.8) |  | 1963 (42.2) |  | .352 | | 0.97 | 0.90 | 1.04 |  |  |  | | |  |  |  |
| Underweight/ normal | 7134 (57.0) |  | 5375 (43.0) |  |  | | 1 |  |  |  |  |  | | |  |  |  |
| Type of delivery |  |  |  |  |  | |  |  |  |  |  |  | | |  |  |  |
| C/S | 4129 (58.1) |  | 2978 (41.9) |  | .536 | | 1.02 | 0.96 | 1.08 |  |  |  | | |  |  |  |
| NSD | 7648 (58.5) |  | 5415 (41.5) |  |  | | 1 |  |  |  |  |  | | |  |  |  |
| Antibiotic use in the first year of life |  |  |  |  |  | |  |  |  |  |  |  | | |  |  |  |
| Yes | 1290 (45.9) |  | 1519 (54.1) |  | <0.001 | | 1.80 | 1.66 | 1.95 |  | <0.001 | 1.45 | | | 1.33 | 1.58 |  |
| No (including unknown) | 10400 (60.4) |  | 6813 (39.6) |  |  | | 1 |  |  |  |  | 1 | | |  |  |  |
| Bronchiolitis before the age of two |  |  |  |  |  | |  |  |  |  |  |  | | |  |  |  |
| Yes | 1427 (43.7) |  | 1842 (56.3) |  | <0.001 | | 2.05 | 1.90 | 2.21 |  | <0.001 | 1.89 | | | 1.74 | 2.05 |  |
| No (including unknown) | 10289 (61.4) |  | 6475 (38.6) |  |  | | 1 |  |  |  |  | 1 | | |  |  |  |
| Birth weight |  |  |  |  |  | |  |  |  |  |  |  | | |  |  |  |
| Underweight (<2500 g) | 725 (58.2) |  | 520 (41.8) |  | .623 | | 0.97 | 0.86 | 1.09 |  |  |  | | |  |  |  |
| Normal (≧2500 g) | 9975 (57.5) |  | 7367 (42.5) |  |  | | 1 |  |  |  |  |  | | |  |  |  |
| Older siblings |  |  |  |  |  | |  |  |  |  |  |  | | |  |  |  |
| Yes | 6328 (64.1) |  | 3537 (35.9) |  | <0.001 | | 0.63 | 0.59 | 0.66 |  | <0.001 | 0.61 | | | 0.57 | 0.65 |  |
| No | 5528 (52.9) |  | 4924 (47.1) |  |  | | 1 |  |  |  |  | 1 | | |  |  |  |
| Younger siblings (703, 2.8%) |  |  |  |  |  | |  |  |  |  |  |  | | |  |  |  |
| Yes | 4045 (55.3) |  | 3266 (44.7) |  | <0.001 | | 1.21 | 1.15 | 1.29 |  | .621 | 0.98 | | | 0.92 | 1.05 |  |
| No | 7811 (60.1) |  | 5195 (39.9) |  |  | | 1 |  |  |  |  | 1 | | |  |  |  |
| Breast- feeding |  |  |  |  |  | |  |  |  |  |  |  | | |  |  |  |
| <4 months (including none) | 9731 (58.4) |  | 6929 (41.6) |  | .926 | | 1.00 | 0.93 | 1.08 |  |  |  | | |  |  |  |
| ≧4 months | 2104 (58.5) |  | 1493 (41.5) |  |  | | 1 |  |  |  |  |  | | |  |  |  |
| Cats in the first year of life |  |  |  |  |  | |  |  |  |  |  |  | | |  |  |  |
| Yes | 233 (51.7) |  | 218 (48.3) |  | .003 | | 1.33 | 1.10 | 1.60 |  | .056 | 1.21 | | | 1.00 | 1.48 |  |
| No | 11696 (58.6) |  | 8254 (41.4) |  |  | | 1 |  |  |  |  | 1 | | |  |  |  |
| Cats in the past 12 months |  |  |  |  |  | |  |  |  |  |  |  | | |  |  |  |
| Yes | 302 (56.6) |  | 232 (43.4) |  | .372 | | 1.08 | 0.91 | 1.29 |  |  |  | | |  |  |  |
| No | 11614 (58.5) |  | 8245 (41.5) |  |  | | 1 |  |  |  |  |  | | |  |  |  |
| Dogs in the first year of life |  |  |  |  |  | |  |  |  |  |  |  | | |  |  |  |
| Yes | 1327 (54.3) |  | 1115 (45.7) |  | <0.001 | | 1.21 | 1.11 | 1.32 |  | .013 | 1.12 | | | 1.02 | 1.23 |  |
| No | 10588 (59.0) |  | 7364 (41.0) |  |  | | 1 |  |  |  |  | 1 | | |  |  |  |
| Dogs in the past 12 months |  |  |  |  |  | |  |  |  |  |  |  | | |  |  |  |
| Yes | 1218 (58.1) |  | 879 (41.9) |  | .721 | | 1.02 | 0.93 | 1.11 |  |  |  | | |  |  |  |
| No | 10701 (58.5) |  | 7595 (41.5) |  |  | | 1 |  |  |  |  |  | | |  |  |  |
| Farm animals in the first year of life |  |  |  |  |  | |  |  |  |  |  |  | | |  |  |  |
| Yes | 227 (50.9) |  | 219 (49.1) |  | .001 | | 1.37 | 1.13 | 1.65 |  | .031 | 1.25 | | | 1.02 | 1.52 |  |
| No | 11687 (58.6) |  | 8259 (41.4) |  |  | | 1 |  |  |  |  | 1 | | |  |  |  |

C/S: Caesarean section; NSD: Normal spontaneous delivery

The p < 0.05 is in univariate analysis.

The multivariate analyses were adjusted for a post-hoc test by dividing the p -value of 0.05 with 8 (the amount of the significant factors in univariate analysis), that is, the corrected p -value is about 0.0063.

## S10 Table. The collinearity analysis for S9 Table.

|  | **Tolerance** | **VIF** |
| --- | --- | --- |
| Gender | .997 | 1.003 |
| Antibiotic use in the first year of life | .910 | 1.099 |
| Bronchiolitis before the age of two | .902 | 1.108 |
| Older siblings | .799 | 1.251 |
| Younger siblings | .807 | 1.240 |
| Cats in the first year of life | .973 | 1.028 |
| Dogs in the first year of life | .982 | 1.019 |
| Farm animals in the first year of life | .967 | 1.034 |

## S11 Table. The relationship between the personal factors and rhinitis symptoms for mild rhinitis in question 1 & 2.

|  | **Not at all**  **(*n* = 1162)** |  | **mild rhinitis**  **(n = 4883)** |  |  |  | **Univariate** | | |  |  | |  | **Multivariate** | | | |
| --- | --- | --- | --- | --- | --- | --- | --- | --- | --- | --- | --- | --- | --- | --- | --- | --- | --- |
| **Variable** | ***n (%)*** |  | ***N*** |  | ***P*** | | ***OR*** | ***lower*** | ***upper*** |  | ***P*** | ***OR*** | | | ***lower*** | ***upper*** |  |
| Gender |  |  |  |  |  | |  |  |  |  |  |  | | |  |  |  |
| Male | 679 (19.7) |  | 2762 (80.3) |  | .247 | | 0.93 | 0.81 | 1.05 |  |  |  | | |  |  |  |
| Female | 483 (18.5) |  | 2121 (81.5) |  |  | | 1 |  |  |  |  |  | | |  |  |  |
| BMI |  |  |  |  |  | |  |  |  |  |  |  | | |  |  |  |
| Overweight/ obesity | 284 (19.5) |  | 1171 (80.5) |  | .350 | | 0.93 | 0.80 | 1.08 |  |  |  | | |  |  |  |
| Underweight/ normal | 689 (18.4) |  | 3057 (81.6) |  |  | | 1 |  |  |  |  |  | | |  |  |  |
| Type of delivery |  |  |  |  |  | |  |  |  |  |  |  | | |  |  |  |
| C/S | 432 (19.9) |  | 1743 (80.1) |  | .305 | | 0.93 | 0.82 | 1.07 |  |  |  | | |  |  |  |
| NSD | 711 (18.8) |  | 3076 (81.2) |  |  | | 1 |  |  |  |  |  | | |  |  |  |
| Antibiotic use in the first year of life |  |  |  |  |  | |  |  |  |  |  |  | | |  |  |  |
| Yes | 171 (16.4) |  | 870 (83.6) |  | .017 | | 1.24 | 1.04 | 1.49 |  | .509 | 1.07 | | | 0.88 | 1.30 |  |
| No (including unknown) | 956 (19.6) |  | 3912 (80.4) |  |  | | 1 |  |  |  |  | 1 | | |  |  |  |
| Bronchiolitis before the age of two |  |  |  |  |  | |  |  |  |  |  |  | | |  |  |  |
| Yes | 215 (15.1) |  | 1208 (84.9) |  | <0.001 | | 1.44 | 1.22 | 1.69 |  | .087 | 1.17 | | | 0.98 | 1.40 |  |
| No (including unknown) | 913 (20.4) |  | 3573 (79.6) |  |  | | 1 |  |  |  |  | 1 | | |  |  |  |
| Birth weight |  |  |  |  |  | |  |  |  |  |  |  | | |  |  |  |
| Underweight (<2500 g) | 72 (18.3) |  | 322 (81.7) |  | .720 | | 1.05 | 0.81 | 1.37 |  |  |  | | |  |  |  |
| Normal (≧2500 g) | 990 (19.0) |  | 4218 (81.0) |  |  | | 1 |  |  |  |  |  | | |  |  |  |
| Older siblings |  |  |  |  |  | |  |  |  |  |  |  | | |  |  |  |
| Yes | 470 (17.9) |  | 2150 (82.1) |  | .033 | | 1.15 | 1.01 | 1.31 |  | .012 | 1.19 | | | 1.04 | 1.37 |  |
| No | 680 (20.1) |  | 2698 (79.9) |  |  | | 1 |  |  |  |  | 1 | | |  |  |  |
| Younger siblings |  |  |  |  |  | |  |  |  |  |  |  | | |  |  |  |
| Yes | 427 (18.8) |  | 1846 (81.2) |  | .552 | | 1.04 | 0.91 | 1.19 |  |  |  | | |  |  |  |
| No | 723 (19.4) |  | 3002 (80.6) |  |  | | 1 |  |  |  |  |  | | |  |  |  |
| Breast- feeding |  |  |  |  |  | |  |  |  |  |  |  | | |  |  |  |
| <4 months (including none) | 220 (20.6) |  | 847 (79.4) |  | .211 | | 1.11 | 0.94 | 1.31 |  |  |  | | |  |  |  |
| ≧4 months | 928 (19.0) |  | 3969 (81.0) |  |  | | 1 |  |  |  |  |  | | |  |  |  |
| Diagnosed asthma |  |  |  |  |  | |  |  |  |  |  |  | | |  |  |  |
| Yes | 116 (11.7) |  | 874 (88.3) |  | <0.001 | | 1.98 | 1.61 | 2.43 |  | <0.001 | 1.88 | | | 1.51 | 2.35 |  |
| No | 1038 (20.8) |  | 3951 (79.2) |  |  | | 1 |  |  |  |  | 1 | | |  |  |  |
| Diagnosed eczema |  |  |  |  |  | |  |  |  |  |  |  | | |  |  |  |
| Yes | 314 (14.8) |  | 1812 (85.2) |  | <0.001 | | 1.62 | 1.40 | 1.87 |  | <0.001 | 1.54 | | | 1.32 | 1.78 |  |
| No | 809 (21.9) |  | 2886 (78.1) |  |  | | 1 |  |  |  |  | 1 | | |  |  |  |
| Cats in the first year of life |  |  |  |  |  | |  |  |  |  |  |  | | |  |  |  |
| Yes | 24 (15.7) |  | 129 (84.3) |  | .267 | | 1.28 | 0.83 | 1.99 |  |  |  | | |  |  |  |
| No | 1129 (19.3) |  | 4729 (80.7) |  |  | | 1 |  |  |  |  |  | | |  |  |  |
| Cats in the past 12 months |  |  |  |  |  | |  |  |  |  |  |  | | |  |  |  |
| Yes | 24 (14.4) |  | 143 (85.6) |  | .110 | | 1.43 | 0.92 | 2.21 |  |  |  | | |  |  |  |
| No | 1131 (19.3) |  | 4718 (80.7) |  |  | | 1 |  |  |  |  |  | | |  |  |  |
| Dogs in the first year of life |  |  |  |  |  | |  |  |  |  |  |  | | |  |  |  |
| Yes | 161 (20.1) |  | 639 (79.9) |  | .464 | | 0.93 | 0.77 | 1.12 |  |  |  | | |  |  |  |
| No | 992 (19.0) |  | 4221 (81.0) |  |  | | 1 |  |  |  |  |  | | |  |  |  |
| Dogs in the past 12 months |  |  |  |  |  | |  |  |  |  |  |  | | |  |  |  |
| Yes | 133 (21.4) |  | 489 (78.6) |  | .141 | | 0.86 | 0.70 | 1.05 |  |  |  | | |  |  |  |
| No | 1021 (18.9) |  | 4373 (81.1) |  |  | | 1 |  |  |  |  |  | | |  |  |  |
| Farm animals in the first year of life |  |  |  |  |  | |  |  |  |  |  |  | | |  |  |  |
| Yes | 26 (17.4) |  | 123 (82.6) |  | .605 | | 1.12 | 0.73 | 1.72 |  |  |  | | |  |  |  |
| No | 1122 (19.1) |  | 4741 (80.9) |  |  | | 1 |  |  |  |  |  | | |  |  |  |

C/S: Caesarean section; NSD: Normal spontaneous delivery

The p < 0.05 is in univariate analysis.

The multivariate analyses were adjusted for a post-hoc test by dividing the p -value of 0.05 with 5 (the amount of the significant factors in univariate analysis), that is, the corrected p -value is 0.01.

## S12 Table. The collinearity analysis for S11 Table.

|  | **Tolerance** | **VIF** |
| --- | --- | --- |
| Antibiotic use in the first year of life | .896 | 1.117 |
| Bronchiolitis before the age of two | .855 | 1.170 |
| Older siblings | .993 | 1.007 |
| Diagnosed asthma | .949 | 1.054 |
| Diagnosed eczema | .968 | 1.033 |

## S13 Table. The relationship between the personal factors and rhinitis symptoms for moderate rhinitis in question 1 & 2.

|  | **Not at all**  **(*n* = 1162)** |  | **moderate rhinitis**  **(n = 3249)** |  |  |  | **Univariate** | | |  |  | |  | **Multivariate** | | | |
| --- | --- | --- | --- | --- | --- | --- | --- | --- | --- | --- | --- | --- | --- | --- | --- | --- | --- |
| **Variable** | ***n (%)*** |  | ***N*** |  | ***P*** | | ***OR*** | ***lower*** | ***upper*** |  | ***P*** | ***OR*** | | | ***lower*** | ***upper*** |  |
| Gender |  |  |  |  |  | |  |  |  |  |  |  | | |  |  |  |
| Male | 679 (26.5) |  | 1883 (73.5) |  | .777 | | 0.98 | 0.86 | 1.12 |  |  |  | | |  |  |  |
| Female | 483 (26.1) |  | 1366 (73.9) |  |  | | 1 |  |  |  |  |  | | |  |  |  |
| BMI |  |  |  |  |  | |  |  |  |  |  |  | | |  |  |  |
| Overweight/ obesity | 284 (26.8) |  | 777 (73.2) |  | .291 | | 0.92 | 0.78 | 1.08 |  |  |  | | |  |  |  |
| Underweight/ normal | 689 (25.1) |  | 2056 (74.9) |  |  | | 1 |  |  |  |  |  | | |  |  |  |
| Type of delivery |  |  |  |  |  | |  |  |  |  |  |  | | |  |  |  |
| C/S | 432 (27.3) |  | 1153 (72.7) |  | .275 | | 0.93 | 0.80 | 1.06 |  |  |  | | |  |  |  |
| NSD | 711 (25.7) |  | 2051 (74.3) |  |  | | 1 |  |  |  |  |  | | |  |  |  |
| Antibiotic use in the first year of life |  |  |  |  |  | |  |  |  |  |  |  | | |  |  |  |
| Yes | 171 (19.2) |  | 718 (80.8) |  | <0.001 | | 1.64 | 1.36 | 1.96 |  | .010 | 1.30 | | | 1.06 | 1.58 |  |
| No (including unknown) | 956 (28.0) |  | 2453 (72.0) |  |  | | 1 |  |  |  |  | 1 | | |  |  |  |
| Bronchiolitis before the age of two |  |  |  |  |  | |  |  |  |  |  |  | | |  |  |  |
| Yes | 215 (18.0) |  | 982 (82.0) |  | <0.001 | | 1.92 | 1.63 | 2.27 |  | <0.001 | 1.40 | | | 1.16 | 1.68 |  |
| No (including unknown) | 913 (29.6) |  | 2172 (70.4) |  |  | | 1 |  |  |  |  | 1 | | |  |  |  |
| Birth weight |  |  |  |  |  | |  |  |  |  |  |  | | |  |  |  |
| Underweight (<2500 g) | 72 (24.2) |  | 226 (75.8) |  | .472 | | 1.11 | 0.84 | 1.46 |  |  |  | | |  |  |  |
| Normal (≧2500 g) | 990 (26.1) |  | 2809 (73.9) |  |  | | 1 |  |  |  |  |  | | |  |  |  |
| Older siblings |  |  |  |  |  | |  |  |  |  |  |  | | |  |  |  |
| Yes | 470 (26.5) |  | 1305 (73.5) |  | .793 | | 0.98 | 0.86 | 1.13 |  |  |  | | |  |  |  |
| No | 680 (26.1) |  | 1923 (73.9) |  |  | | 1 |  |  |  |  |  | | |  |  |  |
| Younger siblings |  |  |  |  |  | |  |  |  |  |  |  | | |  |  |  |
| Yes | 427 (25.3) |  | 1262 (74.7) |  | .240 | | 1.09 | 0.95 | 1.25 |  |  |  | | |  |  |  |
| No | 723 (26.9) |  | 1966 (73.1) |  |  | | 1 |  |  |  |  |  | | |  |  |  |
| Breast- feeding |  |  |  |  |  | |  |  |  |  |  |  | | |  |  |  |
| <4 months (including none) | 928 (26.2) |  | 2619 (73.8) |  | .498 | | 1.06 | 0.89 | 1.26 |  |  |  | | |  |  |  |
| ≧4 months | 220 (27.3) |  | 585 (72.7) |  |  | | 1 |  |  |  |  |  | | |  |  |  |
| Diagnosed asthma |  |  |  |  |  | |  |  |  |  |  |  | | |  |  |  |
| Yes | 116 (13.3) |  | 758 (86.7) |  | <0.001 | | 2.76 | 2.24 | 3.40 |  | <0.001 | 2.27 | | | 1.81 | 2.85 |  |
| No | 1038 (29.7) |  | 2460 (70.3) |  |  | | 1 |  |  |  |  | 1 | | |  |  |  |
| Diagnosed eczema |  |  |  |  |  | |  |  |  |  |  |  | | |  |  |  |
| Yes | 314 (17.9) |  | 1444 (82.1) |  | <0.001 | | 2.24 | 1.93 | 2.60 |  | <0.001 | 2.00 | | | 1.71 | 2.33 |  |
| No | 809 (32.7) |  | 1662 (67.3) |  |  | | 1 |  |  |  |  | 1 | | |  |  |  |
| Cats in the first year of life |  |  |  |  |  | |  |  |  |  |  |  | | |  |  |  |
| Yes | 24 (20.5) |  | 93 (79.5) |  | .151 | | 1.39 | 0.89 | 2.20 |  |  |  | | |  |  |  |
| No | 1129 (26.5) |  | 3137 (73.5) |  |  | | 1 |  |  |  |  |  | | |  |  |  |
| Cats in the past 12 months |  |  |  |  |  | |  |  |  |  |  |  | | |  |  |  |
| Yes | 161 (27.9) |  | 417 (72.1) |  | .101 | | 1.46 | 0.93 | 2.29 |  |  |  | | |  |  |  |
| No | 992 (26.1) |  | 2814 (73.9) |  |  | | 1 |  |  |  |  |  | | |  |  |  |
| Dogs in the first year of life |  |  |  |  |  | |  |  |  |  |  |  | | |  |  |  |
| Yes | 133 (28.8) |  | 329 (71.2) |  | .362 | | 0.91 | 0.75 | 1.11 |  |  |  | | |  |  |  |
| No | 1021 (26.1) |  | 2896 (73.9) |  |  | | 1 |  |  |  |  |  | | |  |  |  |
| Dogs in the past 12 months |  |  |  |  |  | |  |  |  |  |  |  | | |  |  |  |
| Yes | 133 (28.8) |  | 329 (71.2) |  | .209 | | 0.87 | 0.70 | 1.08 |  |  |  | | |  |  |  |
| No | 1021 (26.1) |  | 2896 (73.9) |  |  | | 1 |  |  |  |  |  | | |  |  |  |
| Farm animals in the first year of life |  |  |  |  |  | |  |  |  |  |  |  | | |  |  |  |
| Yes | 26 (22.4) |  | 90 (77.6) |  | .347 | | 1.24 | 0.79 | 1.92 |  |  |  | | |  |  |  |
| No | 1122 (26.3) |  | 3142 (73.7) |  |  | | 1 |  |  |  |  |  | | |  |  |  |

C/S: Caesarean section; NSD: Normal spontaneous delivery

The p < 0.05 is in univariate analysis.

The multivariate analyses were adjusted for a post-hoc test by dividing the p -value of 0.05 with 4 (the amount of the significant factors in univariate analysis), that is, the corrected p -value is 0.0125.

## S14 Table. The collinearity analysis for S13 Table.

|  | **Tolerance** | **VIF** |
| --- | --- | --- |
| Antibiotic use in the first year of life | .915 | 1.092 |
| Bronchiolitis before the age of two | .854 | 1.171 |
| Diagnosed asthma | .912 | 1.097 |
| Diagnosed eczema | .962 | 1.040 |

## S15 Table. The relationship between the personal factors and rhinitis symptoms for severe rhinitis in question 1 & 2.

|  | **Not at all**  **(*n* = 1162)** |  | **severe rhinitis**  **(n = 1024)** |  |  |  | **Univariate** | | |  |  | |  | **Multivariate** | | | |
| --- | --- | --- | --- | --- | --- | --- | --- | --- | --- | --- | --- | --- | --- | --- | --- | --- | --- |
| **Variable** | ***n (%)*** |  | ***N*** |  | ***P*** | | ***OR*** | ***lower*** | ***upper*** |  | ***P*** | ***OR*** | | | ***lower*** | ***upper*** |  |
| Gender |  |  |  |  |  | |  |  |  |  |  |  | | |  |  |  |
| Male | 679 (52.6) |  | 612 (47.4) |  | .527 | | 1.06 | 0.89 | 1.25 |  |  |  | | |  |  |  |
| Female | 483 (54.0) |  | 412 (46.0) |  |  | | 1 |  |  |  |  |  | | |  |  |  |
| BMI |  |  |  |  |  | |  |  |  |  |  |  | | |  |  |  |
| Overweight/ obesity | 284 (53.8) |  | 244 (46.2) |  | .516 | | 0.94 | 0.76 | 1.14 |  |  |  | | |  |  |  |
| Underweight/ normal | 689 (52.1) |  | 633 (47.9) |  |  | | 1 |  |  |  |  |  | | |  |  |  |
| Type of delivery |  |  |  |  |  | |  |  |  |  |  |  | | |  |  |  |
| C/S | 432 (54.7) |  | 358 (45.3) |  | .358 | | 0.92 | 0.77 | 1.10 |  |  |  | | |  |  |  |
| NSD | 711 (52.6) |  | 640 (47.4) |  |  | | 1 |  |  |  |  |  | | |  |  |  |
| Antibiotic use in the first year of life |  |  |  |  |  | |  |  |  |  |  |  | | |  |  |  |
| Yes | 171 (40.8) |  | 248 (59.2) |  | <0.001 | | 1.85 | 1.49 | 2.30 |  | .053 | 1.29 | | | 1.00 | 1.66 |  |
| No (including unknown) | 956 (56.0) |  | 750 (44.0) |  |  | | 1 |  |  |  |  | 1 | | |  |  |  |
| Bronchiolitis before the age of two |  |  |  |  |  | |  |  |  |  |  |  | | |  |  |  |
| Yes | 215 (37.8) |  | 354 (62.2) |  | <0.001 | | 2.33 | 1.91 | 2.84 |  | <0.001 | 1.54 | | | 1.22 | 1.94 |  |
| No (including unknown) | 913 (58.6) |  | 645 (41.4) |  |  | | 1 |  |  |  |  | 1 | | |  |  |  |
| Birth weight |  |  |  |  |  | |  |  |  |  |  |  | | |  |  |  |
| Underweight (<2500 g) | 72 (51.4) |  | 68 (48.6) |  | .715 | | 1.07 | 0.76 | 1.50 |  |  |  | | |  |  |  |
| Normal (≧2500 g) | 990 (53.0) |  | 877 (47.0) |  |  | | 1 |  |  |  |  |  | | |  |  |  |
| Older siblings |  |  |  |  |  | |  |  |  |  |  |  | | |  |  |  |
| Yes | 470 (54.5) |  | 393 (45.5) |  | .344 | | 0.92 | 0.77 | 1.09 |  |  |  | | |  |  |  |
| No | 680 (52.4) |  | 618 (47.6) |  |  | | 1 |  |  |  |  |  | | |  |  |  |
| Younger siblings |  |  |  |  |  | |  |  |  |  |  |  | | |  |  |  |
| Yes | 427 (50.2) |  | 424 (49.8) |  | .023 | | 1.22 | 1.03 | 1.45 |  | .062 | 1.20 | | | 0.99 | 1.46 |  |
| No | 723 (55.2) |  | 587 (44.8) |  |  | | 1 |  |  |  |  | 1 | | |  |  |  |
| Breast- feeding |  |  |  |  |  | |  |  |  |  |  |  | | |  |  |  |
| <4 months (including none) | 928 (52.9) |  | 825 (47.1) |  | .582 | | 1.06 | 0.86 | 1.32 |  |  |  | | |  |  |  |
| ≧4 months | 220 (54.5) |  | 184 (45.5) |  |  | | 1 |  |  |  |  |  | | |  |  |  |
| Diagnosed asthma |  |  |  |  |  | |  |  |  |  |  |  | | |  |  |  |
| Yes | 116 (27.2) |  | 311 (72.8) |  | <0.001 | | 4.03 | 3.19 | 5.09 |  | <0.001 | 3.24 | | | 2.50 | 4.21 |  |
| No | 1038 (60.0) |  | 691 (40.0) |  |  | | 1 |  |  |  |  | 1 | | |  |  |  |
| Diagnosed eczema |  |  |  |  |  | |  |  |  |  |  |  | | |  |  |  |
| Yes | 314 (37.0) |  | 534 (63.0) |  | <0.001 | | 3.11 | 2.59 | 3.72 |  | <0.001 | 2.65 | | | 2.18 | 3.22 |  |
| No | 809 (64.6) |  | 443 (35.4) |  |  | | 1 |  |  |  |  | 1 | | |  |  |  |
| Cats in the first year of life |  |  |  |  |  | |  |  |  |  |  |  | | |  |  |  |
| Yes | 24 (53.3) |  | 21 (46.7) |  | .997 | | 1.00 | 0.55 | 1.81 |  |  |  | | |  |  |  |
| No | 1129 (53.3) |  | 989 (46.7) |  |  | | 1 |  |  |  |  |  | | |  |  |  |
| Cats in the past 12 months |  |  |  |  |  | |  |  |  |  |  |  | | |  |  |  |
| Yes | 24 (57.1) |  | 18 (42.9) |  | .615 | | 0.85 | 0.46 | 1.58 |  |  |  | | |  |  |  |
| No | 1131 (53.2) |  | 994 (46.8) |  |  | | 1 |  |  |  |  |  | | |  |  |  |
| Dogs in the first year of life |  |  |  |  |  | |  |  |  |  |  |  | | |  |  |  |
| Yes | 161 (52.6) |  | 145 (47.4) |  | .808 | | 1.03 | 0.81 | 1.31 |  |  |  | | |  |  |  |
| No | 992 (53.4) |  | 867 (46.6) |  |  | | 1 |  |  |  |  |  | | |  |  |  |
| Dogs in the past 12 months |  |  |  |  |  | |  |  |  |  |  |  | | |  |  |  |
| Yes | 133 (55.0) |  | 109 (45.0) |  | .578 | | 0.93 | 0.71 | 1.21 |  |  |  | | |  |  |  |
| No | 1021 (53.1) |  | 903 (46.9) |  |  | | 1 |  |  |  |  |  | | |  |  |  |
| Farm animals in the first year of life |  |  |  |  |  | |  |  |  |  |  |  | | |  |  |  |
| Yes | 26 (38.8) |  | 41 (61.2) |  | .019 | | 1.81 | 1.10 | 2.99 |  | .032 | 1.93 | | | 1.06 | 3.53 |  |
| No | 1122 (53.5) |  | 975 (46.5) |  |  | | 1 |  |  |  |  |  | | |  |  |  |

C/S: Caesarean section; NSD: Normal spontaneous delivery

The p < 0.05 is in univariate analysis.

The multivariate analyses were adjusted for a post-hoc test by dividing the p -value of 0.05 with 6 (the amount of the significant factors in univariate analysis), that is, the corrected p -value is about 0.0083.

## S16 Table. The collinearity analysis for S15 Table.

|  | **Tolerance** | **VIF** |
| --- | --- | --- |
| Antibiotic use in the first year of life | .876 | 1.142 |
| Bronchiolitis before the age of two | .819 | 1.221 |
| Younger siblings | .830 | 1.206 |
| Diagnosed asthma | .908 | 1.101 |
| Diagnosed eczema | .927 | 1.079 |

## S17 Table. The relationship between the personal factors and rhinitis symptoms for rhinitis ever in question 1.

|  | **No rhinitis**  **(*n* = 11817)** |  | **rhinitis**  **(n = 12100)** |  |  |  | **Univariate** | | |  |  | |  | **Multivariate** | | | |
| --- | --- | --- | --- | --- | --- | --- | --- | --- | --- | --- | --- | --- | --- | --- | --- | --- | --- |
| **Variable** | ***n (%)*** |  | ***N*** |  | ***P*** | | ***OR*** | ***lower*** | ***upper*** |  | ***P*** | ***OR*** | | | ***lower*** | ***upper*** |  |
| Gender |  |  |  |  |  | |  |  |  |  |  |  | | |  |  |  |
| Male | 5506 (44.4) |  | 6892 (55.6) |  | <0.001 | | 1.52 | 1.44 | 1.60 |  | <0.001 | 1.46 | | | 1.38 | 1.55 |  |
| Female | 6311 (54.8) |  | 5208 (45.2) |  |  | | 1 |  |  |  |  | 1 | | |  |  |  |
| BMI |  |  |  |  |  | |  |  |  |  |  |  | | |  |  |  |
| Overweight/ obesity | 2660 (47.8) |  | 2906 (52.2) |  | .360 | | 1.03 | 0.97 | 1.10 |  |  |  | | |  |  |  |
| Underweight/ normal | 7027 (48.5) |  | 7458 (51.5) |  |  | | 1 |  |  |  |  |  | | |  |  |  |
| Type of delivery |  |  |  |  |  | |  |  |  |  |  |  | | |  |  |  |
| C/S | 4090 (48.8) |  | 4286 (51.2) |  | .267 | | 1.03 | 0.98 | 1.09 |  |  |  | | |  |  |  |
| NSD | 7493 (49.6) |  | 7618 (50.4) |  |  | | 1 |  |  |  |  |  | | |  |  |  |
| Antibiotic use in the first year of life |  |  |  |  |  | |  |  |  |  |  |  | | |  |  |  |
| Yes | 1304 (36.1) |  | 2306 (63.9) |  | <0.001 | | 1.90 | 1.77 | 2.04 |  | <0.001 | 1.37 | | | 1.26 | 1.49 |  |
| No (including unknown) | 10194 (51.8) |  | 9490 (48.2) |  |  | | 1 |  |  |  |  | 1 | | |  |  |  |
| Bronchiolitis before the age of two |  |  |  |  |  | |  |  |  |  |  |  | | |  |  |  |
| Yes | 1546 (33.0) |  | 3144 (67.0) |  | <0.001 | | 2.35 | 2.19 | 2.51 |  | <0.001 | 1.73 | | | 1.60 | 1.87 |  |
| No (including unknown) | 9959 (53.6) |  | 8636 (46.4) |  |  | | 1 |  |  |  |  | 1 | | |  |  |  |
| Birth weight |  |  |  |  |  | |  |  |  |  |  |  | | |  |  |  |
| Underweight (<2500 g) | 727 (47.5) |  | 802 (52.5) |  | .392 | | 1.05 | 0.94 | 1.16 |  |  |  | | |  |  |  |
| Normal (≧2500 g) | 9819 (48.7) |  | 10351 (51.3) |  |  | | 1 |  |  |  |  |  | | |  |  |  |
| Older siblings |  |  |  |  |  | |  |  |  |  |  |  | | |  |  |  |
| Yes | 6311 (54.9) |  | 5186 (45.1) |  | <0.001 | | 0.65 | 0.61 | 0.68 |  | <0.001 | 0.64 | | | 0.60 | 0.68 |  |
| No | 5347 (44.0) |  | 6802 (56.0) |  |  | | 1 |  |  |  |  | 1 | | |  |  |  |
| Younger siblings |  |  |  |  |  | |  |  |  |  |  |  | | |  |  |  |
| Yes | 3921 (46.0) |  | 4596 (54.0) |  | <0.001 | | 1.23 | 1.16 | 1.29 |  | .877 | 0.99 | | | 0.93 | 1.06 |  |
| No | 7737 (51.1) |  | 7392 (48.9) |  |  | | 1 |  |  |  |  | 1 | | |  |  |  |
| Breast- feeding |  |  |  |  |  | |  |  |  |  |  |  | | |  |  |  |
| <4 months (including none) | 9542 (49.4) |  | 9772 (50.6) |  | .851 | | 0.99 | 0.93 | 1.06 |  |  |  | | |  |  |  |
| ≧4 months | 2087 (49.2) |  | 2151 (50.8) |  |  | | 1 |  |  |  |  |  | | |  |  |  |
| Diagnosed asthma |  |  |  |  |  | |  |  |  |  |  |  | | |  |  |  |
| Yes | 800 (25.7) |  | 2314 (74.3) |  | <0.001 | | 3.28 | 3.01 | 3.57 |  | <0.001 | 2.43 | | | 2.22 | 2.67 |  |
| No | 10920 (53.1) |  | 9637 (46.9) |  |  | | 1 |  |  |  |  | 1 | | |  |  |  |
| Diagnosed eczema |  |  |  |  |  | |  |  |  |  |  |  | | |  |  |  |
| Yes | 2605 (35.8) |  | 4680 (64.2) |  | <0.001 | | 2.30 | 2.17 | 2.44 |  | <0.001 | 1.94 | | | 1.82 | 2.06 |  |
| No | 8836 (56.2) |  | 6898 (43.8) |  |  | | 1 |  |  |  |  | 1 | | |  |  |  |
| Cats in the first year of life |  |  |  |  |  | |  |  |  |  |  |  | | |  |  |  |
| Yes | 229 (43.0) |  | 304 (57.0) |  | .003 | | 1.30 | 1.10 | 1.55 |  | .032 | 1.24 | | | 1.02 | 1.50 |  |
| No | 11506 (49.6) |  | 11707 (50.4) |  |  | | 1 |  |  |  |  | 1 | | |  |  |  |
| Cats in the past 12 months |  |  |  |  |  | |  |  |  |  |  |  | | |  |  |  |
| Yes | 293 (47.2) |  | 328 (52.8) |  | .266 | | 1.09 | 0.93 | 1.28 |  |  |  | | |  |  |  |
| No | 11431 (49.4) |  | 11689 (50.6) |  |  | | 1 |  |  |  |  |  | | |  |  |  |
| Dogs in the first year of life |  |  |  |  |  | |  |  |  |  |  |  | | |  |  |  |
| Yes | 1304 (44.8) |  | 1609 (55.2) |  | <0.001 | | 1.23 | 1.14 | 1.33 |  | .038 | 1.10 | | | 1.01 | 1.20 |  |
| No | 10413 (50.0) |  | 10415 (50.0) |  |  | | 1 |  |  |  |  | 1 | | |  |  |  |
| Dogs in the past 12 months |  |  |  |  |  | |  |  |  |  |  |  | | |  |  |  |
| Yes | 1188 (48.4) |  | 1266 (51.6) |  | .324 | | 1.04 | 0.96 | 1.13 |  |  |  | | |  |  |  |
| No | 10529 (49.5) |  | 10758 (50.5) |  |  | | 1 |  |  |  |  |  | | |  |  |  |
| Farm animals in the first year of life | 219 (39.5) |  | 336 (60.5) |  |  | |  |  |  |  |  |  | | |  |  |  |
| Yes | 1150049.6) |  | 1168350.4) |  | <0.001 | | 1.51 | 1.27 | 1.79 |  | .017 | 1.27 | | | 1.04 | 1.54 |  |
| No | 12614 (55.0) |  | 10300 (45.0) |  |  | | 1 |  |  |  |  | 1 | | |  |  |  |

C/S: Caesarean section; NSD: Normal spontaneous delivery

The p < 0.05 is in univariate analysis.

The multivariate analyses were adjusted for a post-hoc test by dividing the p -value of 0.05 with 10 (the amount of the significant factors in univariate analysis), that is, the corrected p -value is about 0.005.

## S18 Table. The collinearity analysis for S17 Table.

|  | **Tolerance** | **VIF** |
| --- | --- | --- |
| Gender | .994 | 1.006 |
| Antibiotic use in the first year of life | .898 | 1.114 |
| Bronchiolitis before the age of two | .843 | 1.186 |
| Older siblings | .987 | 1.013 |
| Diagnosed asthma | .923 | 1.084 |
| Diagnosed eczema | .955 | 1.047 |
| Cats in the first year of life | .981 | 1.020 |
| Dogs in the first year of life | .964 | 1.037 |
| Farm animals in the first year of life | .982 | 1.019 |

**S19 Table. The relationship between effect and cause in literature.**

| **Effect** | **Cause** | **Reference** |
| --- | --- | --- |
| Antibiotic use in the first year of life | Gender | no |
|  | Type of delivery | no |
|  | Birth weight | no |
|  | Older siblings | no |
|  | Farm animals in the first year of life | no |
| Bronchiolitis before the age of two | Gender | [45] |
|  | Type of delivery | [46] |
|  | Antibiotic use in the first year of life | no |
|  | Birth weight | [45] |
|  | Older siblings | [45] |
|  | Farm animals in the first year of life | no |
| Diagnosed asthma | Gender | [12,13,14] |
|  | BMI | [38] |
|  | Type of delivery | [38] |
|  | Antibiotic use in the first year of life | [6,25] |
|  | Bronchiolitis before the age of two | [44] |
|  | Birth weight | [38] |
|  | Breast- feeding | [38] |
|  | Diagnosed eczema | [1] |
|  | Dogs in the first year of life | [39] |
|  | Farm animals in the first year of life | [40] |
| Diagnosed eczema | BMI | [41] |
|  | Antibiotic use in the first year of life | [25] |
|  | Bronchiolitis before the age of two | [43] |
|  | Older siblings | [42] |
|  | Younger siblings | no |
|  | Breast- feeding | [42] |
|  | Diagnosed asthma | [1] |
|  | Cats in the first year of life | [39] |
|  | Dogs in the first year of life | [39] |
|  | Farm animals in the first year of life | no |
| **AR** | Gender | [11-13] |
|  | Antibiotic use in the first year of life | [2,20-23] |
|  | Bronchiolitis before the age of two | no |
|  | Older siblings | [30-32] |
|  | Diagnosed asthma | [1,2,8,14,34-36] |
|  | Diagnosed asthma | [1,2,8,14,34-36] |
|  | Cats in the first year of life | [35,39] |

**References for S19 Table (continued to text)**

38. Nascimento JXPT, Ribeiro CCC, Batista RFL, de Britto MTSS, Simões VMF, Padilha LL, et al. The first 1000 days of life factors associated with “childhood asthma symptoms”: Brisa Cohort, Brazil. Scientific reports. 2017;7(1):1-12. <https://doi.org/10.1038/s41598-017-16295-4> PMCID: [PMC5700095](http://www.ncbi.nlm.nih.gov/pmc/articles/pmc5700095/)

39. Luo S, Sun Y, Hou J, Kong X, Wang P, Zhang Q, et al. Pet keeping in childhood and asthma and allergy among children in Tianjin area, China. PloS one. 2018;13(5): e0197274. https://doi.org/[10.1371/journal.pone.0197274](https://doi.org/10.1371/journal.pone.0197274) PMCID: [PMC5955563](http://www.ncbi.nlm.nih.gov/pmc/articles/pmc5955563/)

40. Salam MT, Li YF, Langholz B, Gilliland FD. Early-life environmental risk factors for asthma: findings from the Children's Health Study. Environmental health perspectives. 2004;112(6):760-765. <https://doi.org/10.1289/ehp.6662> PMCID: [PMC1241973](http://www.ncbi.nlm.nih.gov/pmc/articles/pmc1241973/)

41. Pan CX, Jee YH, Moore KJ, Kraft P, Asgari MM. Relationship Between Body Mass Index and Atopic Dermatitis: Critical Appraisal of a Mendelian Randomization Approach in Exploring Causality. Br J Dermatol. 2020. <https://doi.org/10.1111/bjd.19702>

42. Fotopoulou M, Iordanidou M, Vasileiou E, Trypsianis G, Chatzimichael A, Paraskakis E. A short period of breastfeeding in infancy, excessive house cleaning, absence of older sibling, and passive smoking are related to more severe atopic dermatitis in children. Eur J Dermatol. 2018;28(1):56-63. <https://doi.org/10.1684/ejd.2017.3165>

43. Balekian DS, Linnemann RW, Castro VM, Perlis R, Thadhani R, Camargo Jr CA. Pre‐birth cohort study of atopic dermatitis and severe bronchiolitis during infancy. Pediatr Allergy Immunol. 2016;27(4):413-418. <https://doi.org/10.1111/pai.12532>

44. Törmänen S, Lauhkonen E, Riikonen R, Koponen P, Huhtala H, et al. Risk factors for asthma after infant bronchiolitis. Allergy. 2018;73(4):916-922. <https://doi.org/10.1111/all.13347>

45. Nagayama Y, Tsubaki T, Nakayama S, Sawada K, Taguchi K, et al. Gender analysis in acute bronchiolitis due to respiratory syncytial virus. Pediatr Allergy Immunol. 2006;17(1):29-36. <https://doi.org/10.1542/peds.2005-2119>

46. Shang X, Liabsuetrakul T, Sangsupawanich P, Xia X, He P, Cao H. Elective cesarean delivery as a predisposing factor of respiratory syncytial virus bronchiolitis in children. J Med Assoc Thai. 2014;97(8):827-34. PMID: [25345258](https://pubmed.ncbi.nlm.nih.gov/25345258/)
